# Association between *KCNQ1* gene polymorphisms and gestational diabetes mellitus susceptibility in a Chinese population

**DOI:** 10.3389/fendo.2025.1451942

**Published:** 2025-07-02

**Authors:** Yanying Wu, Yuxuan Zhang, Xin Liu, Jia Liu, Zhaotao He, Yue Wei, Qiaoli Zeng, Runmin Guo

**Affiliations:** ^1^ Department of Internal Medicine, Shunde Women and Children’s Hospital (Maternity and Child Healthcare Hospital of Shunde Foshan), Guangdong Medical University, Foshan, Guangdong, China; ^2^ Key Laboratory of Research in Maternal and Child Medicine and Birth Defects, Guangdong Medical University, Foshan, Guangdong, China; ^3^ Maternal and Child Research Institute, Shunde Women and Children’s Hospital (Maternity and Child Healthcare Hospital of Shunde Foshan), Guangdong Medical University, Foshan, Guangdong, China; ^4^ Department of Ultrasound, Shunde Women and Children’s Hospital (Maternity and Child Healthcare Hospital of Shunde Foshan), Guangdong Medical University, Foshan, Guangdong, China

**Keywords:** gestational diabetes mellitus, potassium voltage-gated channel subfamily Q member 1, single nucleotide polymorphism, rs2237897, rs163184, rs151290, rs2237892

## Abstract

**Introduction:**

The potassium voltage-gated channel subfamily Q member 1 (*KCNQ1*) gene is recognized as a type 2 diabetes mellitus (T2DM) susceptibility gene. However, there is limited data regarding the association between KCNQ1 gene polymorphisms and gestational diabetes mellitus (GDM) susceptibility in China. To explore the association between *KCNQ1* gene polymorphisms and GDM susceptibility in a Chinese population.

**Methods:**

We conducted a case-control study including 500 pregnant women with GDM and 502 pregnant women with normal glucose tolerance (as controls). Blood samples and clinical data were collected. *KCNQ1* gene rs2237897, rs163184, rs151290, and rs2237892 were genotyped by SNPscan™ genotyping assay. Using SPSS V.26.0, statistical analysis was performed to explore the association of *KCNQ1* gene polymorphisms with GDM and genotypes with blood glucose levels. Meta-analysis was further validated in different populations.

**Results:**

After being adjusted for confounding factors (age, parity, pre-pregnancy BMI (pre-BMI) and blood pressure) and Bonferroni correction, rs2237897 showed an association with decreased GDM risk in codominant heterozygous (CT vs. CC: OR = 0.537; 95% CI: 0.354-0.816; *P* = 0.004) and overdominant models (CT vs. CC+TT: OR = 0.533; 95% CI: 0.355-0.801; *P* = 0.002) in pregnant women aged < 30 years. However, rs2237892, rs151290, and rs163184 did not found associations with GDM after Bonferroni correction. Meta-analysis showed that rs2237892 was associated with decreased GDM risk in different races in dominant (TC+TT vs. CC: OR = 0.830; 95% CI: 0.699-0.985; *P* = 0.033), recessive (TT vs. CT+CC: OR = 0.733; 95% CI: 0.612-0.877; *P* = 0.001), codominant homozygous (TT vs. CC: OR = 0.679; 95% CI: 0.562-0.820; *P* < 0.001), codominant heterozygous (TC vs. CC: OR = 0.843; 95% CI: 0.753-0.945; *P* = 0.003) and allele models (T vs. C: OR = 0.852; 95% CI: 0.740-0.982; *P* = 0.027).

**Conclusion:**

*KCNQ1* rs2237897 is associated with decreased GDM risk in a Chinese population. Although rs2237892 did not found association with GDM risk in our subjects, meta-analysis confirmed that rs2237892 is associated with reduced GDM risk across different populations. Further studies are needed to confirm these findings and elucidate the mechanisms.

## 1 Introduction

Gestational diabetes mellitus (GDM) is a disorder of glucose tolerance first identified during pregnancy, and its prevalence continues to increase around the world (1, 2). A global observational study reported that the prevalence of GDM varied from 9.3% to 25.5%, with an overall prevalence of 17.8% (3). GDM can cause perinatal complications such as gestational hypertension and preeclampsia, as well as lead to adverse pregnancy outcomes like abortion, preterm birth, macrosomia, neonatal respiratory distress syndrome, and neonatal hypoglycemia (4). Although the exact pathogenesis of GDM remains unclear, evidence suggests that it involves a complex interplay of genetic, environmental, and metabolic factors. Single nucleotide polymorphisms (SNPs), common genetic variations in the human genome, have been implicated in the susceptibility to certain diseases, including type 2 diabetes mellitus (T2DM) and GDM. Genes associated with T2DM susceptibility and involved in pancreatic beta cell function, insulin sensitivity, and glucose regulation are potential candidate genes for GDM (5).

The potassium voltage-gated channel subfamily Q member 1 (*KCNQ1*) gene, located on chromosome 11 (11p15.5), encodes the α subunit of the voltage gated K^+^ channel (Kv7.1) and is expressed in the human pancreas. This channel plays a critical role in insulin secretion. Inhibition of the *KCNQ1* gene in pancreatic beta cells can increase insulin secretion and insulin granules exocytosis, whereas overexpression decreases insulin exocytosis and secretion, thereby enhancing T2DM susceptibility (6, 7). A genome-wide association study (GWAS) in a Japanese population first identified *KCNQ1* as a risk gene for T2DM, and then subsequently confirmed in Chinese, Koreans, Swedes, and Danes (8–14). The studies on *KCNQ1* polymorphisms and GDM risk have been conducted in various populations, including Chinese, Korean, Japanese, Saudi, Mexican, Pakistani, and Caucasian (15–23). However, in China, research on KCNQ1 polymorphisms has primarily focused on T2DM, with few studies on GDM. Moreover, research on GDM and genetic polymorphisms in the Chinese population has mainly focused on rs2237892, with no reports on rs2237897, rs151290, and rs163184. Against this backdrop, we aimed to investigate the association of KCNQ1 gene polymorphisms (rs2237897, rs163184, rs151290, and rs2237892) with GDM risk in a Chinese population, providing valuable theoretical insights for the early detection and prevention of GDM.

## 2 Materials and methods

### 2.1 Study subjects

From 1 August 2021 to 31 January 2022, the study recruited 1002 participants at Shunde Women and Children’s Hospital, Guangdong Medical University. The subjects included in this study are the same as those studied by Zeng et al. (24).

According to the diagnostic criteria of the International Association of Diabetes and Pregnancy Study Groups (IADPSG), all pregnant women underwent a 75g oral glucose tolerance test (OGTT) at 24–28 weeks of gestation, measuring plasma glucose at fasting, 1 hour, and 2 hours. The OGTT was conducted in the morning following an 8-hour fasting period. GDM was diagnosed if glucose levels exceeded any of the following thresholds: fasting of 92 mg/dL (5.1 mmol/L), 1-hour of 180 mg/dL (10.0 mmol/L), 2-hour of 153 mg/dL (8.5 mmol/L) (25). Based on the results of OGTT, we divided the pregnant women into a case group with GDM and a control group with normal glucose tolerance.

Exclusion criteria were: aged < 18 years; not Han nationality; patients with a previous history of hyperthyroidism, diabetes, Cushing’s syndrome, or other conditions affecting blood glucose levels; patients with hypertension, hepatic insufficiency, renal insufficiency, severe cardiovascular disease, or pregnancy complications; patients taking hypoglycemic drugs; participants unable to participate in the study or unwilling to provide written informed consent. After exclusion, we included 1002 pregnant women (500 cases with GDM and 502 controls without diabetes) in *KCNQ1* rs163184, rs151290, and rs2237892 and 1000 pregnant women (500 cases with GDM and 500 controls without diabetes) in *KCNQ1* rs2237897.

The participants provided their informed consent, and the study received Ethics Committee approval of Shunde Women and Children’s Hospital of Guangdong Medical University, adhering to the guidelines of the Helsinki Declaration.

### 2.2 Data collection

The study involved the collection of clinical and biochemical data from the participants, including age, pre-pregnancy height and weight, blood pressure, blood glucose levels, parity, neonatal weight, and gestational age. The calculation of pre-pregnancy body mass index (pre-BMI) followed the formula: BMI (kg/m^2^) =weight (kg)/height^2^ (m^2^).

### 2.3 SNP genotyping

Genomic DNA was extracted from two ml peripheral venous blood of pregnant women using the QIAamp DNA Blood Kit (Qiagen, Germany). The SNPscan™ genotyping assay (Genesky Technologies Inc., Shanghai, China) was utilized to genotype the SNPs.

### 2.4 Statistical analyses

Statistical analyses were conducted using SPSS V.26.0. Categorical variables were presented as frequencies and percentages, while continuous numerical variables were expressed as “mean ± standard deviation”. Differences in baseline characteristics between the case and control groups were compared using the independent samples t-test and chi-square test. The chi-square test was also used to evaluate Hardy-Weinberg equilibrium (HWE) in the control group. Genotype and allele frequencies for each SNP were determined. Logistic regression analysis was employed to investigate the association between *KCNQ1* genetic polymorphisms and the risk of GDM, utilizing six genetic models: dominant, recessive, overdominant, allele, codominant homozygous, and codominant heterozygous. Adjusted odds ratios (ORs) and 95% confidence intervals (CIs) were calculated to quantify the relationship between *KCNQ1* polymorphisms and GDM risk, adjusting for potential confounders including age, parity, pre-pregnancy body mass index (pre-BMI), diastolic blood pressure, and systolic blood pressure. Stratified analyses by age and pre-BMI were performed to further explore the relationship between genetic polymorphisms and GDM risk. One-way ANOVA was applied to assess the correlation between genotype and continuous outcomes such as blood glucose levels, gestational age, and neonatal weight. Linkage disequilibrium (LD) and haplotype analyses were performed using the SHEsis.plus platform (http://shesisplus.bio-x.cn/SHEsis.html), excluding haplotypes with frequencies below 0.03. The significance level of α = 0.05 was chosen, and *P* < 0.05 were considered statistically significant. The Bonferroni correction was applied to account for multiple testing, specifically considering the number of independent genetic variants, which adjusted the significance threshold to α < 0.0125 (calculated as 0.05 divided by 4).

### 2.5 Meta-analyses

**Figure 1:**
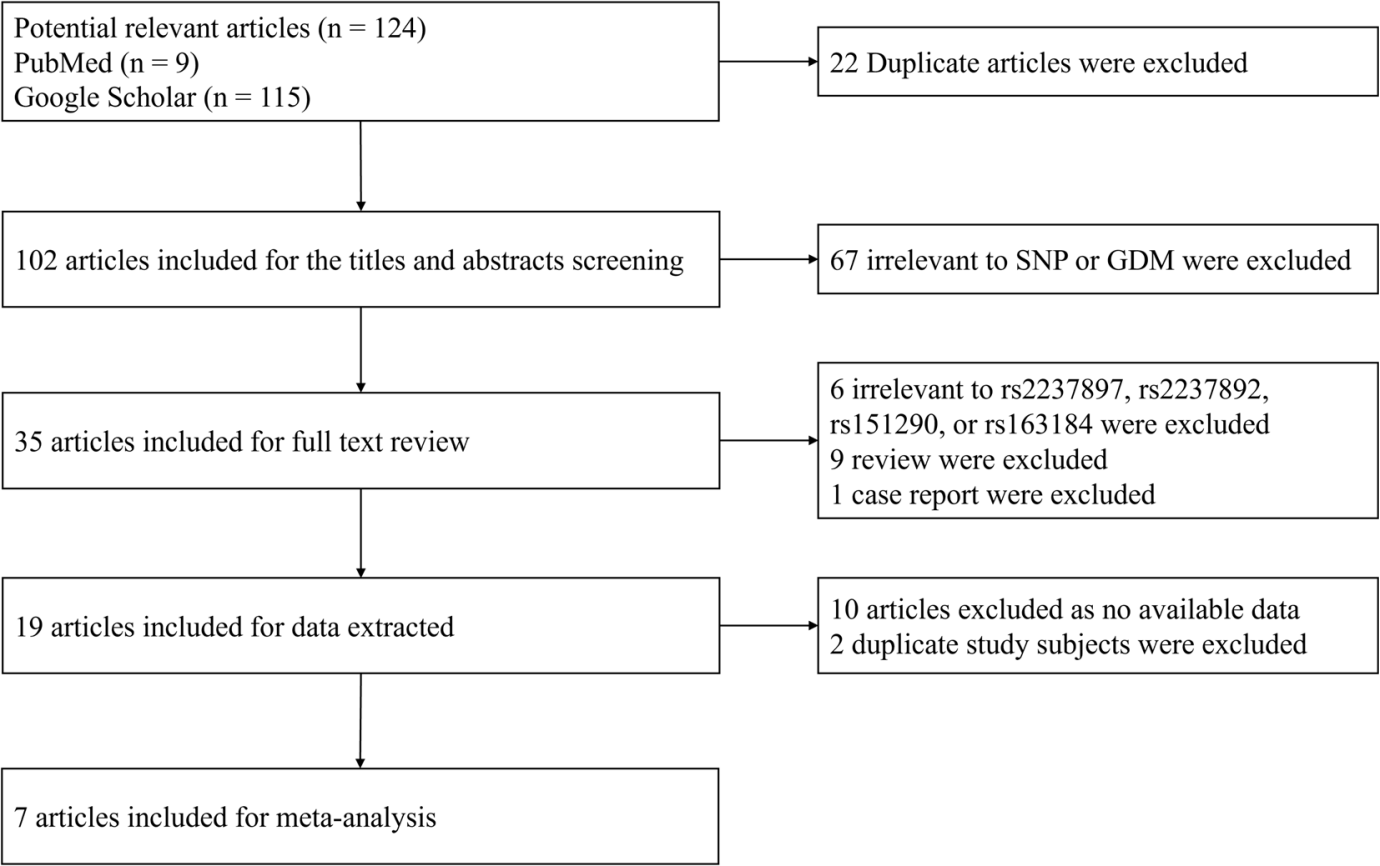
Flow chart of the literature search and selection.

A systematic literature search was performed using PubMed and Google Scholar databases for articles containing the terms rs2237897, rs163184, rs151290, rs2237892, and gestational diabetes mellitus (GDM) (**Figure 1**). The inclusion criteria were: (a) original papers, (b) case-control or cohort studies, and (c) sufficient raw data, including the frequency of genotype distributions, OR values, and 95% CIs. Studies were excluded if they did not adhere to diagnostic criteria, had data deviating from Hardy-Weinberg equilibrium, presented overlapping data, or were purely case or family studies. A total of seven studies on rs2237892 and GDM susceptibility (including our own) (16–21) and three studies on rs151290 and GDM susceptibility (including our own) (21, 26) were selected. Six genetic models were analyzed using either a fixed effects model or a random effects model based on the level of heterogeneity. Funnel plots were used to assess publication bias, and Egger’s test and Begg’s test were employed to evaluate heterogeneity. STATA V.16.0 software was applied for the meta-analysis.

## 3 Results

### 3.1 General characteristics

We conducted a case-control study that included 500 subjects with GDM in the case group and 502 healthy subjects in the control group. For the *KCNQ1* rs163184, rs151290, and rs2237892, the mean age, pre-BMI, systolic blood pressure, diastolic blood pressure, fasting blood glucose, 1-hour OGTT blood glucose, and 2-hour OGTT blood glucose levels were significantly higher in the case group compared to the control group (*P* < 0.05). Additionally, the chi-squared results showed a significant difference in parity between the two groups (*P* < 0.05), with a higher number of multiparas in the case group. For rs2237897, the mean age, pre-BMI, systolic blood pressure, diastolic blood pressure, fasting blood glucose, 1-hour OGTT blood glucose, and 2-hour OGTT blood glucose of the case group were significantly higher than those of the control group (*P <* 0.05). There was a significant difference in the parity, with more multiparous women in the case group (**Supplementary Table 1**). According to Chinese BMI classification, individuals are categorized as underweight if their BMI is less than 18.5 kg/m^2^, normal weight if their BMI is between 18.5 and 24 kg/m^2^, overweight if their BMI is between 24 and 28 kg/m^2^, and obese if their BMI is more than 28 kg/m^2^ (27, 28). Subjects were classified into three groups: underweight with a pre-BMI less than 18.5 kg/m^2^, normal weight with a pre-BMI between 18.5 and 24 kg/m^2^, and overweight with a pre-BMI more than 24 kg/m^2^. For rs163184, rs151290, and rs2237892, there was a significant difference in pre-BMI between the case and control groups (*P* < 0.05), with approximately 70% of pregnant women having a pre-BMI ranging from 18.5 to 24 kg/m^2^. The number of pregnant women with a pre-BMI ≥ 24 kg/m^2^ was significantly higher in the case group (**Table 1**). For rs2237897, the pre-BMI of the two groups was significantly different (*P* < 0.05), with a great number of pregnant women having a pre-BMI ≥ 24 kg/m^2^ in the case group (**Supplementary Table 1**). Aged ≥ 30 years is one of the risk factors for GDM (29). Pregnant women were divided into two groups, aged < 30 years and aged ≥ 30 years, and it was found that there were more pregnant women aged ≥ 30 years in the case group.

**Table 1:** Basic and stratified characteristic of participants with *KCNQ1* rs163184, rs151290, rs2237892.

Variables	NGT (n = 502)	GDM (n = 500)	t/χ^2^	*P*
Age (year)	29 ± 4	31 ± 4	-8.562	**< 0.001**
Pre-BMI (kg/m^2^)	20.53 ± 2.58	21.51 ± 3.10	-5.415	**< 0.001**
SBP (mmHg)	114 ± 10	117 ± 11	-3.528	**< 0.001**
DBP (mmHg)	68 ± 7	70 ± 8	-3.231	**0.001**
FBG (mmol/L)	4.50 ± 0.31	4.82 ± 0.64	-9.745	**< 0.001**
1h-PG (mmol/L)	7.66 ± 1.26	10.17 ± 1.60	-26.222	**< 0.001**
2h-PG (mmol/L)	6.69 ± 0.99	8.91 ± 1.59	-25.850	**< 0.001**
Age (year)			49.200	**< 0.001**
< 30	304 (0.606)	192 (0.384)		
≥ 30	198 (0.394)	308 (0.616)		
Pre-BMI (kg/m^2^)			27.798	**< 0.001**
< 18.5	95 (0.189)	67 (0.134)		
18.5 ≤ pre-BMI < 24	365 (0.727)	336 (0.672)		
≥24	42 (0.084)	97 (0.194)		
Parity (n)			8.882	**0.003**
0	258 (0.514)	210 (0.42)		
≥ 1	244 (0.486)	290 (0.58)		

NGT, normal glucose tolerance; GDM, Gestational diabetes mellitus; Pre-BMI, pre-gestational body mass index; SBP, systolic blood pressure; DBP, diastolic blood pressure; FBG, fasting blood glucose level; 1h-PG, 1 hour blood glucose level; 2h-PG, 2 hour blood glucose level; bold values indicate the *P* < 0.05.

### 3.2 Association of rs2237897, rs163184, rs151290 and rs2237892 with GDM

#### 3.2.1 Overall analysis results

The minor allele frequencies of rs2237897, rs163184, rs151290, and rs2237892 were 0.287, 0.434, 0.375, and 0.295. All SNPs were located within the intronic region of the *KCNQ1* gene. Additionally, each SNP in the control group was in Hardy-Weinberg equilibrium (*P >* 0.05) (**Supplementary Table 2**). After adjusting for confounding factors (age, parity, pre-BMI, diastolic and systolic blood pressure), rs2237897 showed an association with decreased GDM risk in codominant heterozygous (CT vs. CC: OR = 0.725; 95% CI: 0.549-0.957; *P* = 0.023), dominant (CT+TT vs. CC: OR = 0.744; 95% CI: 0.572-0.968; *P* = 0.027) and overdominant models (CT vs. TT+CC: OR = 0.749; 95% CI: 0.573-0.980; *P* = 0.035). Rs151290 showed an association with decreased GDM risk in the overdominant model (CA vs. CC+AA: OR = 0.764; 95% CI: 0.587-0.994; *P* = 0.045). Rs2237892 was linked to the decreased risk of GDM in codominant heterozygous (TC vs. CC: OR = 0.745; 95% CI: 0.565-0.983; *P* = 0.038) and overdominant models (TC vs. TT+CC: OR = 0.754; 95% CI: 0.577-0.985; *P* = 0.038). However, these associations lost statistical significance after Bonferroni correction for multiple testing (adjusted significance threshold *P* < 0.0125). No significant association was observed for rs163184 across any genetic models (**Table 2**).

**Table 2:** The associations between *KCNQ1* gene and GDM risk in overall subjects.

Model	Controls (%)	Cases (%)	Crude OR (95% CI)	Crude *P*	Adjusted OR (95% CI)	Adjusted *P*
rs2237897						
Codominant model						
CC	242 (0.484)	267 (0.534)	1 (Reference)		1 (Reference)	
CT	212 (0.424)	195 (0.390)	0.834 (0.642-1.082)	0.172	0.725 (0.549-0.957)	0.023
TT	46 (0.092)	38 (0.076)	0.749 (0.471-1.190)	0.221	0.805 (0.497-1.306)	0.380
Dominant Model						
CC	242 (0.484)	267 (0.534)	1 (Reference)		1 (Reference)	
CT+TT	258 (0.516)	233 (0.466)	0.819 (0.639-1.049)	0.114	0.744 (0.572-0.968)	0.027
Recessive Model						
CT+CC	454 (0.908)	462 (0.924)	1 (Reference)		1 (Reference)	
TT	46 (0.092)	38 (0.076)	0.812 (0.518-1.272)	0.362	0.940 (0.588-1.502)	0.796
Overdominant model						
TT+CC	288 (0.576)	305 (0.610)	1 (Reference)		1 (Reference)	
CT	212 (0.424)	195 (0.390)	0.869 (0.675-1.118)	0.274	0.749 (0.573-0.980)	0.035
Allele model						
C	696 (0.696)	729 (0.729)	1 (Reference)		1 (Reference)	
T	304 (0.304)	271 (0.271)	0.851 (0.701-1.033)	0.103	0.826 (0.674-1.013)	0.067
rs163184						
Codominant model						
TT	153 (0.305)	150 (0.3)	1 (Reference)		1 (Reference)	
GT	268 (0.534)	260 (0.52)	0.990 (0.746-1.313)	0.942	0.959 (0.711-1.293)	0.784
GG	81 (0.161)	90 (0.18)	1.133 (0.779-1.649)	0.513	1.174 (0.789-1.746)	0.429
Dominant Model						
TT	153 (0.305)	150 (0.3)	1 (Reference)		1 (Reference)	
GT+GG	349 (0.695)	350 (0.7)	1.023 (0.781-1.339)	0.869	1.007 (0.758-1.338)	0.963
Recessive Model						
GT+TT	421 (0.839)	410 (0.82)	1 (Reference)		1 (Reference)	
GG	81 (0.161)	90 (0.18)	1.141 (0.821-1.586)	0.433	1.187 (0.837-1.682)	0.337
Overdominant model						
GG+TT	234 (0.466)	240 (0.48)	1 (Reference)		1 (Reference)	
GT	268 (0.534)	260 (0.52)	0.946 (0.738-1.212)	0.660	0.913 (0.703-1.186)	0.487
Allele model						
T	574 (0.572)	560 (0.56)	1 (Reference)		1 (Reference)	
G	430 (0.428)	440 (0.44)	1.049 (0.879-1.252)	0.597	1.053 (0.874-1.268)	0.587
rs151290						
Codominant model						
CC	190 (0.379)	213 (0.426)	1 (Reference)		1 (Reference)	
CA	239 (0.476)	208 (0.416)	0.776 (0.593-1.017)	0.066	0.756 (0.569-1.005)	0.054
AA	73 (0.145)	79 (0.158)	0.965 (0.664-1.402)	0.853	0.975 (0.654-1.452)	0.899
Dominant Model						
CC	190 (0.379)	213 (0.426)	1 (Reference)		1 (Reference)	
CA+AA	312 (0.621)	287 (0.574)	0.821 (0.637-1.057)	0.125	0.803 (0.615-1.048)	0.106
Recessive Model						
CA+CC	429 (0.855)	421 (0.842)	1 (Reference)		1 (Reference)	
AA	73 (0.145)	79 (0.158)	1.103 (0.781-1.558)	0.579	1.110 (0.773-1.596)	0.572
Overdominant model						
AA+CC	263 (0.524)	292 (0.584)	1 (Reference)		1 (Reference)	
CA	239 (0.476)	208 (0.416)	0.784 (0.611-1.006)	0.056	0.764 (0.587-0.994)	0.045
Allele model						
C	619 (0.617)	634 (0.634)	1 (Reference)		1 (Reference)	
A	385 (0.383)	366 (0.366)	0.928 (0.775-1.112)	0.419	0.920 (0.760-1.113)	0.389
rs2237892						
Codominant model						
CC	240 (0.478)	263 (0.526)	1 (Reference)		1 (Reference)	
TC	215 (0.428)	192 (0.384)	0.815 (0.627-1.059)	0.125	0.745 (0.565-0.983)	0.038
TT	47 (0.094)	45 (0.09)	0.874 (0.560-1.363)	0.552	0.927 (0.580-1.480)	0.750
Dominant Model						
CC	240 (0.478)	263 (0.526)	1 (Reference)		1 (Reference)	
CT+TT	262 (0.522)	237 (0.474)	0.825 (0.644-1.058)	0.130	0.782 (0.602-1.015)	0.065
Recessive Model						
CT+CC	455 (0.906)	455 (0.91)	1 (Reference)		1 (Reference)	
TT	47 (0.094)	45 (0.09)	0.957 (0.623-1.470)	0.842	1.076 (0.685-1.692)	0.750
Overdominant model						
TT+CC	287 (0.572)	308 (0.616)	1 (Reference)		1 (Reference)	
CT	215 (0.428)	192 (0.384)	0.832 (0.646-1.071)	0.154	0.754 (0.577-0.985)	0.038
Allele model						
C	695 (0.692)	718 (0.718)	1 (Reference)		1 (Reference)	
T	309 (0.308)	282 (0.282)	0.883 (0.729-1.071)	0.206	0.876 (0.715-1.072)	0.198

Adjusted *P*-value were calculated using logistic regression, with adjustments made for age, pre-BMI, SBP, DBP and parity. Bolded values denote statistical significance at the *P* < 0.0125. The control group for the SNP rs2237897 comprised 500 participants, while the control groups for SNP rs163184, rs151290, and rs2237892 each consisted of 502 participants. Correspondingly, the case groups for these SNPs (rs2237897, rs163184, rs151290, and rs2237892) each included 500 participants.

#### 3.2.2 Stratified analysis results

The stratified analysis demonstrated that, among pregnant women aged <30 years, the rs2237897 polymorphism exhibited protective effects against GDM across multiple genetic models. In unadjusted analyses, rs2237897 showed an association with the decreased GDM risk in codominant heterozygous (CT vs. CC: OR = 0. 569; 95% CI: 0.380-0.852; *P* = 0.006), dominant (CT+TT vs. CC: OR = 0.660; 95% CI: 0.457-0.952; *P* = 0.026) and overdominant models (CT vs. CC+TT: OR = 0.566; 95% CI: 0.383-0.836; *P* = 0.004). After adjusting for confounding factors (age, parity, pre-BMI, diastolic and systolic blood pressure), rs2237897 showed a significant association with decreased GDM risk in codominant heterozygous (CT vs. CC: OR = 0.537; 95% CI: 0.354-0.816; *P* = 0.004), dominant (CT+TT vs. CC: OR = 0.625; 95% CI: 0.427-0.915; *P* = 0.016) and overdominant models (CT vs. CC+TT: OR = 0.533; 95% CI: 0.355-0.801; *P* = 0.002). Additionally, rs2237897 was associated with decreased GDM risk in codominant heterozygous and overdominant models after Bonferroni correction (**Table 3**). Similarly, in unadjusted analyses, rs2237892 was linked to the decreased risk of GDM in codominant heterozygous (CT vs. CC: OR = 0.624; 95% CI: 0.420-0.926; *P* = 0.019), dominant (CT+TT vs. CC: OR = 0.678; 95% CI: 0.471-0.976; *P* = 0.036), and overdominant models (CT vs. CC+TT: OR = 0.635; 95% CI: 0.433-0.931; *P* = 0.020) in the pregnant women aged < 30 years. Following adjustment for confounding factors (age, parity, pre-BMI, diastolic and systolic blood pressure), rs2237892 was linked to the decreased risk of GDM in codominant heterozygous (CT vs. CC: OR = 0.594; 95% CI: 0.394-0.895; *P* = 0.013), dominant (CT+TT vs. CC: OR = 0.661; 95% CI: 0.453-0.966; *P* = 0.032), and overdominant models (CT vs. CC+TT: OR = 0.605; 95% CI: 0.407-0.901; *P* = 0.013), but no association was found after Bonferroni correction (**Table 3**).

**Table 3:** The associations between *KCNQ1* gene and GDM risk in age < 30 years subjects.

Model	Controls (%)	Cases (%)	Crude OR (95% CI)	Crude *P*	Adjusted OR (95% CI)	Adjusted *P*
rs2237897						
Codominant model						
CC	152 (0.502)	116 (0.604)	1 (Reference)		1 (Reference)	
CT	122 (0.402)	53 (0.276)	0.569 (0.380-0.852)	**0.006**	0.537 (0.354-0.816)	**0.004**
TT	29 (0.096)	23 (0.120)	1.039 (0.571-1.890)	0.9	1.014 (0.544-1.889)	0.966
Dominant Model						
CC	152 (0.502)	116 (0.604)	1 (Reference)		1 (Reference)	
CT+TT	151 (0.498)	76 (0.396)	0.660 (0.457-0.952)	0.026	0.625 (0.427-0.915)	0.016
Recessive Model						
CT+CC	274 (0.904)	169 (0.880)	1 (Reference)		1 (Reference)	
TT	29 (0.096)	23 (0.120)	1.286 (0.720-2.296)	0.395	1.283 (0.701-2.347)	0.419
Overdominant model						
TT+CC	181 (0.598)	139 (0.724)	1 (Reference)		1 (Reference)	
CT	122 (0.402)	53 (0.276)	0.566 (0.383-0.836)	**0.004**	0.533 (0.355-0.801)	**0.002**
Allele model						
C	426 (0.703)	285 (0.742)	1 (Reference)		1 (Reference)	
T	180 (0.297)	99 (0.258)	0.822 (0.617-1.096)	0.182	0.796 (0.591-1.071)	0.132
rs163184						
Codominant model						
TT	94 (0.309)	53 (0.276)	1 (Reference)		1 (Reference)	
GT	161 (0.530)	103 (0.536)	1.135 (0.747-1.723)	0.553	1.054 (0.683-1.627)	0.811
GG	49 (0.161)	36 (0.188)	1.303 (0.755-2.250)	0.342	1.354 (0.756-2.426)	0.308
Dominant Model						
TT	94 (0.309)	53 (0.276)	1 (Reference)		1 (Reference)	
GT+GG	210 (0.691)	139 (0.724)	1.174 (0.788-1.750)	0.431	1.115 (0.737-1.687)	0.606
Recessive Model						
GT+TT	255 (0.839)	156 (0.812)	1 (Reference)		1 (Reference)	
GG	49 (0.161)	36 (0.188)	1.201 (0.748-1.929)	0.449	1.300 (0.792-2.134)	0.299
Overdominant model						
GG+TT	143 (0.470)	89 (0.464)	1 (Reference)		1 (Reference)	
GT	161 (0.530)	103 (0.536)	1.028 (0.716-1.477)	0.882	0.942 (0.646-1.374)	0.757
Allele model						
T	349 (0.574)	209 (0.544)	1 (Reference)		1 (Reference)	
G	259 (0.426)	175 (0.456)	1.128 (0.872-1.459)	0.358	1.128 (0.864-1.472)	0.376
rs151290						
Codominant model						
CC	120 (0.395)	84 (0.438)	1 (Reference)		1 (Reference)	
CA	141 (0.464)	78 (0.406)	0.790 (0.534-1.170)	0.240	0.794 (0.528-1.193)	0.266
AA	43 (0.141)	30 (0.156)	0.997 (0.579-1.716)	0.990	1.068 (0.603-1.891)	0.821
Dominant Model						
CC	120 (0.395)	84 (0.438)	1 (Reference)		1 (Reference)	
CA+AA	184 (0.605)	108 (0.562)	0.839 (0.581-1.209)	0.346	0.845 (0.578-1.237)	0.387
Recessive Model						
CA+CC	261 (0.859)	162 (0.844)	1 (Reference)		1 (Reference)	
AA	43 (0.141)	30 (0.156)	1.124 (0.678-1.864)	0.650	1.118 (0.662-1.888)	0.677
Overdominant model						
AA+CC	163 (0.536)	114 (0.594)	1 (Reference)		1 (Reference)	
CA	141 (0.464)	78 (0.406)	0.791 (0.549-1.140)	0.209	0.800 (0.547-1.169)	0.248
Allele model						
C	381 (0.627)	246 (0.641)	1 (Reference)		1 (Reference)	
A	227 (0.373)	138 (0.359)	0.942 (0.722-1.228)	0.657	0.945 (0.717-1.243)	0.684
rs2237892						
Codominant model						
CC	148 (0.487)	112 (0.583)	1 (Reference)		1 (Reference)	
TC	125 (0.411)	59 (0.307)	0.624 (0.420-0.926)	0.019	0.594 (0.394-0.895)	0.013
TT	31 (0.102)	21 (0.110)	0.895 (0.488-1.641)	0.720	0.927 (0.493-1.745)	0.815
Dominant Model						
CC	148 (0.487)	112 (0.583)	1 (Reference)		1 (Reference)	
CT+TT	156 (0.513)	80 (0.417)	0.678 (0.471-0.976)	0.036	0.661 (0.453-0.966)	0.032
Recessive Model						
CT+CC	273 (0.898)	171 (0.890)	1 (Reference)		1 (Reference)	
TT	31 (0.102)	21 (0.110)	1.081 (0.602-1.943)	0.793	1.139 (0.618-2.098)	0.676
Overdominant model						
TT+CC	179 (0.589)	133 (0.693)	1 (Reference)		1 (Reference)	
CT	125 (0.411)	59 (0.307)	0.635 (0.433-0.931)	0.020	0.605 (0.407-0.901)	0.013
Allele model						
C	421 (0.692)	283 (0.737)	1 (Reference)		1 (Reference)	
T	187 (0.308)	101 (0.263)	0.803 (0.604-1.069)	0.133	0.802 (0.597-1.077)	0.142

Adjusted *P*-values were calculated using logistic regression, accounting for age, pre-BMI, SBP, DBP and parity; bold values indicate the *P* < 0.0125. In the age subgroup of less than 30 years, the control group for SNP rs2237897 consisted of 303 participants, while the control groups for SNPs rs163184, rs151290, and rs2237892 each comprised 304 participants. Similarly, the case groups for these SNPs (rs2237897, rs163184, rs151290, and rs2237892) each contained 192 participants within the same age subgroup.

Among the pregnant women with a pre-BMI ≥ 24 kg/m^2^, rs2237897 showed an association with decreased GDM risk in codominant heterozygous (CT vs. CC: OR = 0.418; 95% CI: 0.192-0.912; *P* = 0.028), dominant (CT+TT vs. CC: OR = 0.415; 95% CI: 0.195-0.884; *P* = 0.023), and allele models (T vs. C: OR = 0.564; 95% CI: 0.327-0.973; *P* = 0.040) in unadjusted analyses, but no association was found after adjusting for confounding factors and Bonferroni correction (**Table 4**). In unadjusted analyses, Rs151290 was found to be linked to the decreased risk of GDM in codominant heterozygous (AC vs. CC: OR = 0.431; 95% CI: 0.192-0.966; *P* = 0.041) and overdominant models (AC vs. CC+AA: OR = 0.423; 95% CI: 0.202-0.886; *P* = 0.023) among the pregnant women with a pre-BMI ≥ 24 kg/m^2^. After adjusting for confounding factors (age, parity, pre-BMI, diastolic and systolic blood pressure), only the overdominant model (AC vs. CC+AA: OR = 0.396; 95% CI: 0.177-0.885; *P* = 0.024) showed an association with the decreased risk of GDM, but no association was found after Bonferroni correction (**Table 4**). No other subgroups showed an association with GDM. (**Supplementary Tables 3**-**5**).

**Table 4:** The associations between *KCNQ1* gene and GDM risk in pre-BMI ≥ 24 kg/m^2^ subjects.

Model	Controls (%)	Cases (%)	Crude OR (95% CI)	Crude *P*	Adjusted OR (95% CI)	Adjusted *P*
rs2237897						
Codominant model						
CC	14 (0.333)	53 (0.546)	1 (Reference)		1 (Reference)	
CT	24 (0.572)	38 (0.392)	0.418 (0.192-0.912)	0.028	0.457 (0.200-1.044)	0.063
TT	4 (0.095)	6 (0.062)	0.396 (0.098-1.600)	0.194	0.785 (0.141-4.369)	0.783
Dominant Model						
CC	14 (0.333)	53 (0.546)	1 (Reference)		1 (Reference)	
CT+TT	28 (0.667)	44 (0.454)	0.415 (0.195-0.884)	0.023	0.485 (0.218-1.080)	0.077
Recessive Model						
CT+CC	38 (0.905)	91 (0.938)	1 (Reference)		1 (Reference)	
TT	4 (0.095)	6 (0.062)	0.626 (0.167-2.346)	0.487	1.343 (0.292-6.183)	0.705
Overdominant model						
TT+CC	18 (0.428)	59 (0.608)	1 (Reference)		1 (Reference)	
CT	24 (0.572)	38 (0.392)	0.483 (0.232-1.007)	0.052	0.451 (0.203-1.000)	0.050
Allele model						
C	52 (0.619)	144 (0.742)	1 (Reference)		1 (Reference)	
T	32 (0.381)	50 (0.258)	0.564 (0.327-0.973)	0.040	0.700 (0.391-1.254)	0.230
rs163184						
Codominant model						
TT	14 (0.333)	30 (0.309)	1 (Reference)		1 (Reference)	
GT	21 (0.5)	54 (0.557)	1.200 (0.534-2.698)	0.659	1.278 (0.528-3.094)	0.587
GG	7 (0.167)	13 (0.134)	0.867 (0.284-2.647)	0.802	0.710 (0.178-2.834)	0.628
Dominant Model						
TT	14 (0.333)	30 (0.309)	1 (Reference)		1 (Reference)	
GT+GG	28 (0.667)	67 (0.691)	1.117 (0.516-2.418)	0.780	1.058 (0.465-2.407)	0.893
Recessive Model						
GT+TT	35 (0.833)	84 (0.866)	1 (Reference)		1 (Reference)	
GG	7 (0.167)	13 (0.134)	0.774 (0.285-2.103)	0.615	0.444 (0.141-1.393)	0.164
Overdominant model						
GG+TT	21 (0.5)	43 (0.443)	1 (Reference)		1 (Reference)	
GT	21 (0.5)	54 (0.557)	1.256 (0.608-2.594)	0.538	1.533 (0.691-3.399)	0.293
Allele model						
T	49 (0.583)	114 (0.588)	1 (Reference)		1 (Reference)	
G	35 (0.417)	80 (0.412)	0.982 (0.584-1.652)	0.947	0.855 (0.493-1.484)	0.579
rs151290						
Codominant model						
CC	13 (0.310)	44 (0.454)	1 (Reference)		1 (Reference)	
CA	24 (0.571)	35 (0.361)	0.431 (0.192-0.966)	0.041	0.489 (0.206-1.162)	0.105
AA	5 (0.119)	18 (0.185)	1.064 (0.331-3.421)	0.918	2.153 (0.527-8.792)	0.286
Dominant Model						
CC	13 (0.310)	44 (0.454)	1 (Reference)		1 (Reference)	
CA+AA	29 (0.690)	53 (0.546)	0.540 (0.251-1.162)	0.115	0.637 (0.282-1.441)	0.279
Recessive Model						
CA+CC	37 (0.881)	79 (0.815)	1 (Reference)		1 (Reference)	
AA	5 (0.119)	18 (0.185)	1.686 (0.581-4.891)	0.336	2.550 (0.788-8.257)	0.118
Overdominant model						
AA+CC	18 (0.429)	62 (0.639)	1 (Reference)		1 (Reference)	
CA	24 (0.571)	35 (0.361)	0.423 (0.202-0.886)	0.023	0.396 (0.177-0.885)	0.024
Allele model						
C	50 (0.595)	123 (0.634)	1 (Reference)		1 (Reference)	
A	34 (0.405)	71 (0.366)	0.849 (0.502-1.434)	0.540	1.031 (0.588-1.810)	0.914
rs2237892						
Codominant model						
CC	15 (0.357)	51 (0.526)	1 (Reference)		1 (Reference)	
TC	23 (0.548)	38 (0.392)	0.486 (0.224-1.054)	0.068	0.590 (0.261-1.334)	0.205
TT	4 (0.095)	8 (0.082)	0.588 (0.155-2.227)	0.435	0.911 (0.196-4.227)	0.905
Dominant Model						
CC	15 (0.357)	51 (0.526)	1 (Reference)		1 (Reference)	
CT+TT	27 (0.643)	46 (0.474)	0.501 (0.238-1.057)	0.070	0.611 (0.277-1.345)	0.221
Recessive Model						
CT+CC	38 (0.905)	89 (0.918)	1 (Reference)		1 (Reference)	
TT	4 (0.095)	8 (0.082)	0.854 (0.242-3.007)	0.806	1.207 (0.289-5.035)	0.797
Overdominant model						
TT+CC	19 (0.452)	59 (0.608)	1 (Reference)		1 (Reference)	
CT	23 (0.548)	38 (0.392)	0.532 (0.256-1.106)	0.091	0.583 (0.268-1.270)	0.174
Allele model						
C	53 (0.631)	140 (0.722)	1 (Reference)		1 (Reference)	
T	31 (0.369)	54 (0.278)	0.659 (0.383-1.135)	0.133	0.786 (0.440-1.404)	0.415

Pre-BMI, pre-gestational body mass index; adjusted *P* value calculated by logistic regression with adjustment for age, pre-BMI, SBP, DBP and parity; bold values indicate the *P* < 0.0125. In the subgroup defined by a pre-BMI ≥ 24 kg/m^2^, the control groups for SNPs rs2237897, rs163184, rs151290, and rs2237892 each comprised 42 participants. Correspondingly, the case groups for these SNPs all included 97 participants within the same pre-BMI subgroup.

### 3.3 Linkage disequilibrium analyses and haplotype analyses

**Figure 2:**
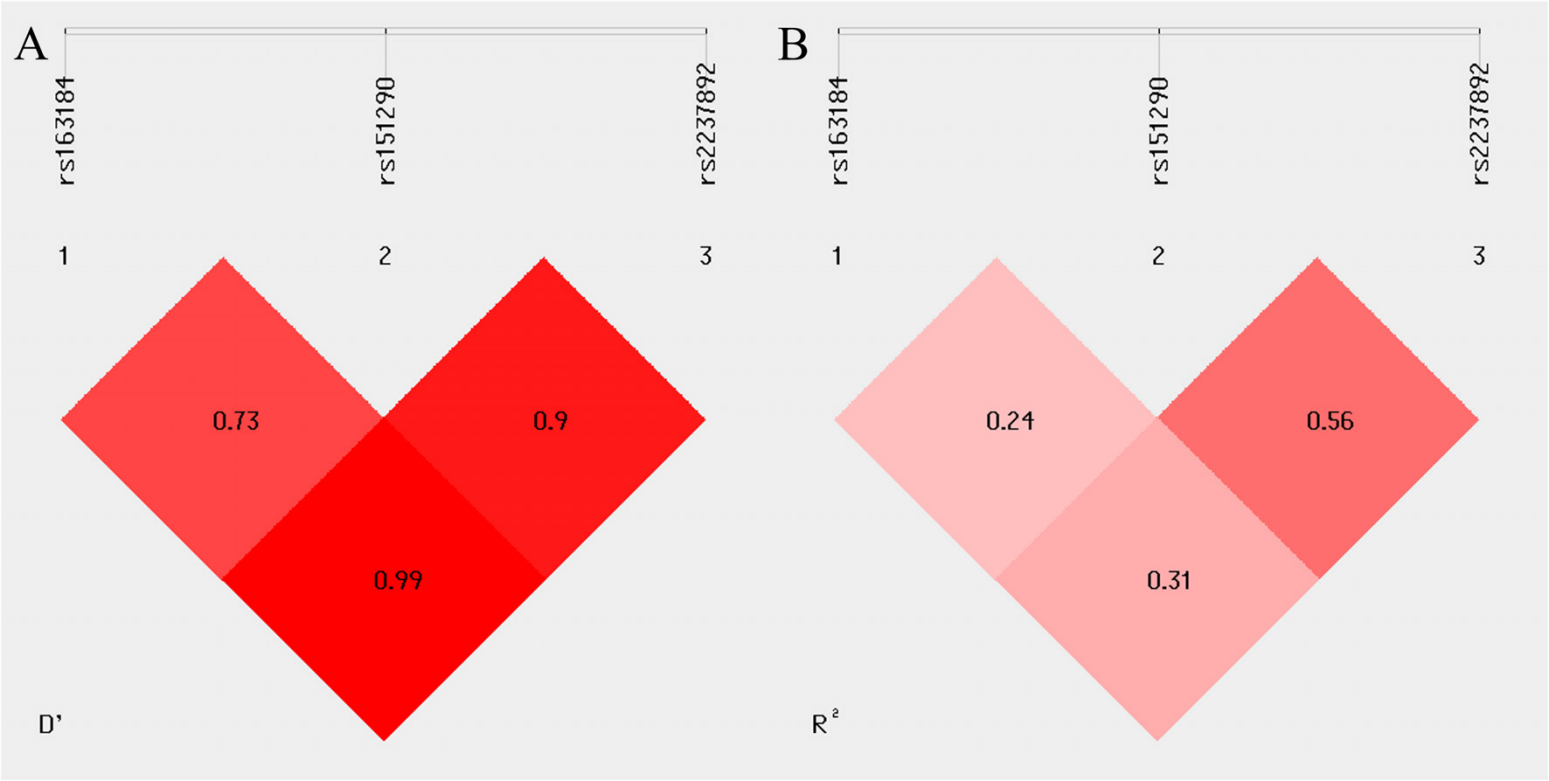
Linkage disequilibrium (LD) between multiple loci of the *KCNQ1* gene (rs163184, rs151290 and rs2237892). **(A)** coefficient of linkage disequilibrium D’; **(B)** correlation coefficient R^2^.

Linkage disequilibrium was observed between rs2237892 and rs163184 (D’ = 0.99, R^2^ = 0.31), rs2237892 and rs151290 (D’ = 0.9, R^2^ = 0.56), rs163184 and rs151290 (D’ = 0.73, R^2^ = 0.24) (**Figure 2**). Haplotype analysis revealed no association with GDM risk, excluding haplotypes with frequencies below 0.03 (*P* > 0.05) (**Supplementary Table 6**).

### 3.4 Associations of blood glucose level, neonatal weight, and gestational age with genotype

The study examined the associations of blood glucose levels, neonatal weight, and gestational age with genotype using ANOVA. However, no significant differences were found (*P* > 0.05) (**Supplementary Table 7**).

### 3.5 Meta-analysis results

**Figure 3:**
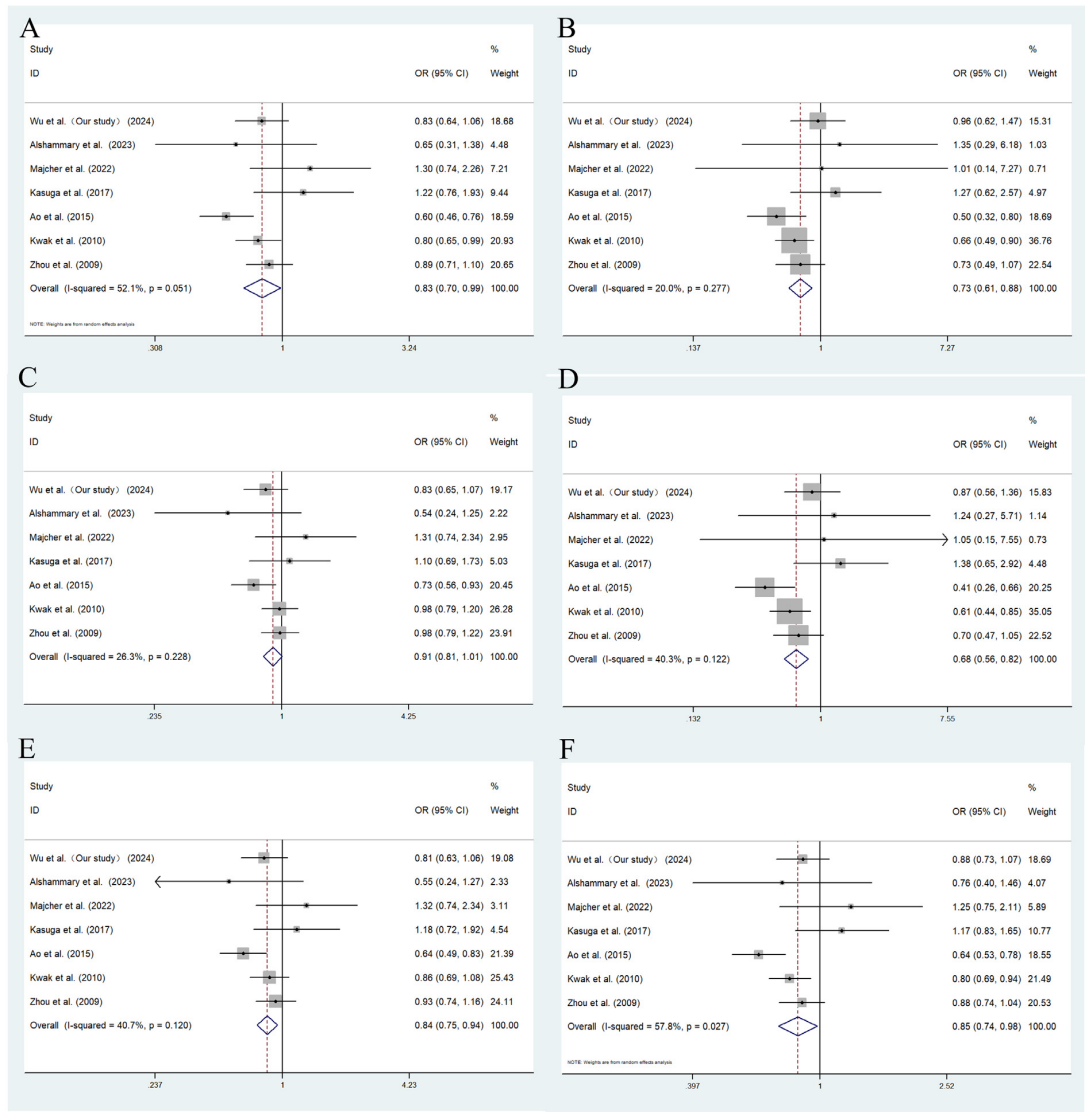
Meta-analysis of the association between *KCNQ1* rs2237892 and GDM susceptibility. **(A)** dominant model, TT+CT vs. CC; **(B)** recessive model, TT vs. CT+CC; **(C)** overdominant model, CT vs. TT+CC; **(D)** codominant homozygous model, TT vs. CC; **(E)** codominant heterozygous model, CT vs. CC; **(F)** allele model, T vs. **(C)** OR, odds ratio; CI, confidence interval; I-squared, measure to quantify the degree of heterogeneity in meta-analyses.

**Figure 4:**
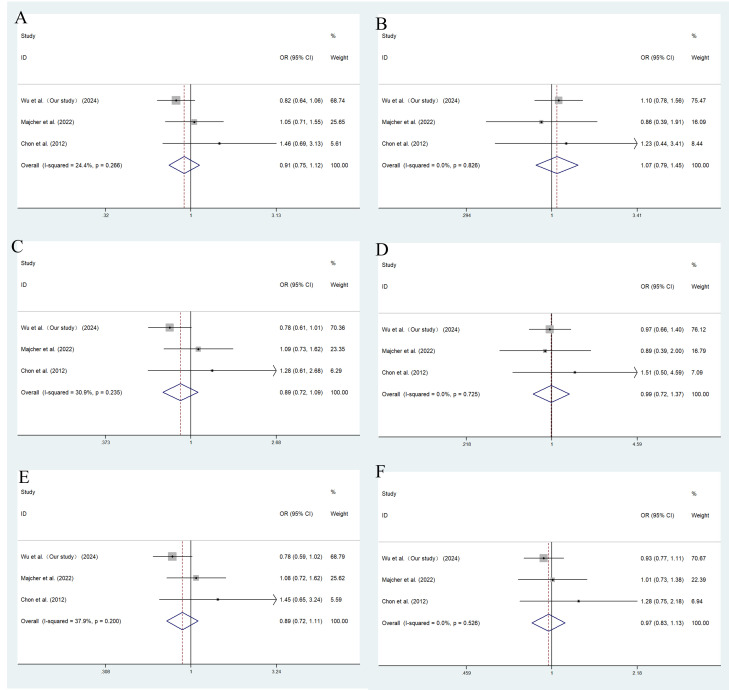
Meta-analysis of the association between *KCNQ1* rs151290 and GDM susceptibility. **(A)** dominant model, AA+CA vs. CC; **(B)** recessive model, AA vs. CA+CC; **(C)** overdominant model, CA vs. AA +CC; **(D)** codominant homozygous model, AA vs. CC; **(E)** codominant heterozygous model, CA vs. CC; **(F)** allele model, A vs. **(C)** OR, odds ratio; CI, confidence interval; I-squared, measure to quantify the degree of heterogeneity in meta-analyses.

A total of 7 studies of rs2237892 with GDM susceptibility (including ours) and 3 studies of rs151290 with GDM susceptibility (including ours) were chosen for meta-analysis. The basic details of these studies were presented in **Supplementary Table 8**. Depending on the degree of heterogeneity, we used the random effects model when I^2^ exceeded 50% and the fixed effects model when I^2^ was below 50%. Egger’s and Begg’s test demonstrated no significant publication bias (*P >* 0.05). Funnel plots were utilized to detect the potential occurrence of publication bias. The shape of the funnel plot is symmetrical, indicating the absence of significant publication bias (**Supplementary Figures 1**, **2**). In dominant (TC+TT vs. CC: OR = 0.830; 95% CI: 0.699-0.985; *P* = 0.033), recessive (TT vs. CT+CC: OR = 0.733; 95% CI: 0.612-0.877; *P* = 0.001), codominant homozygous (TT vs. CC: OR = 0.679; 95% CI: 0.562-0.820; *P* < 0.001), codominant heterozygous (TC vs. CC: OR = 0.843; 95% CI: 0.753-0.945; *P* = 0.003) and allele models (T vs. C: OR = 0.852; 95% CI: 0.740-0.982; *P* = 0.027), rs2237892 show an association with decreased GDM risk in different races (**Figure 3**). However, no association with GDM was found in rs151290 (**Figure 4**) (**Table 5**).

**Table 5:** Result summary of genetic association studies of SNPs and their genetic model.

Genetic model	OR (95% CI), *P <* 0.05	Heterogeneity		meta-analysis model
		Q	I^2^	
rs2237897				
Dominant model	0.830 (0.699-0.985), ***P* = 0.033**	*P* = 0.051	52.10%	random
Recessive model	0.733 (0.612-0.877), ***P* = 0.001**	*P* = 0.277	20.00%	fix
Overdominant model	0.905 (0.812-1.009), *P* = 0.071	*P* = 0.228	26.30%	fix
Codominant homozygous model	0.679 (0.562-0.820), ***P <* 0.001**	*P* = 0.122	40.30%	fix
Codominant heterozygous model	0.843 (0.753-0.945), ***P* = 0.003**	*P* = 0.120	40.70%	fix
Allele model	0.852 (0.740-0.982), ***P* = 0.027**	*P* = 0.027	57.80%	random
rs151290				
Dominant model	0.915 (0.746-1.122), *P* = 0.392	*P* = 0.266	24.40%	fix
Recessive model	1.075 (0.794-1.454), *P* = 0.641	*P* = 0.826	0.00%	fix
Overdominant model	0.886 (0.723-1.086), *P* = 0.244	*P* = 0.235	30.90%	fix
Codominant homozygous model	0.991 (0.717-1.370), *P* = 0.956	*P* = 0.725	0.00%	fix
Codominant heterozygous model	0.891 (0.718-1.106), *P* = 0.294	*P* = 0.200	37.90%	fix
Allele model	0.970 (0.834-1.127), *P* = 0.690	*P* = 0.526	0.00%	fix

I^2^, I-squared variation in OR attributable to heterogeneity; Q, heterogeneity chi-squared *P* value; bold values indicate significant association.

## 4 Discussion

The *KCNQ1* rs2237897 variant, located in an intronic region, has been associated with protective effects against GDM (30). This finding is consistent with a study conducted in Mexico, which identified the TTT haplotype of *KCNQ1* (rs2237897, rs163184, rs2237892) as protective against GDM (22). However, a study conducted in a Chinese population found no association between *KCNQ1* (rs2237892, rs2237897, rs163184) and GDM risk (31). The previous studies revealed that *KCNQ1* rs2237897 was a susceptibility gene for T2DM and was associated with GDM susceptibility, particularly in Asian populations, which aligned with our findings. Our results show that rs2237897 was associated with decreased GDM risk in codominant heterozygous and overdominant models among pregnant women under 30 years old. These findings suggest that the TC genotype and T allele at rs2237897 may have protective effects against GDM, potentially modulated by age. However, the limited number of studies on the association between rs2237897 and GDM, along with some inconsistencies in the literature, suggests that the variant’s effects may vary across populations, underscoring the need for a multicenter study to further validate these findings and clarify the role of rs2237897 in GDM susceptibility.

In both Korean and Caucasian populations, no significant association was observed between GDM risk and the *KCNQ1* rs151290 variant (21, 26). Our study similarly found no association between rs151290 and GDM. Consistent with these findings, our meta-analysis also did not show any association between rs151290 and GDM. The lack of association may be attributed to several factors. Firstly, the genetic effect of rs151290 on GDM susceptibility might vary across different populations, emphasizing the importance of replicating genetic associations in diverse populations. Secondly, rs151290 may exert a minimal effect on GDM risk, requiring larger sample sizes or more powered studies to detect its potential influence. Thirdly, it is possible that rs151290 is not a potential risk variant.

*KCNQ1* rs2237892 showed a significant association with GDM and was linked to 1-hour and 2-hour OGTT glucose levels in Chinese, Korean, and Mexican populations (15, 16, 18, 22). Another study revealed that *KCNQ1* rs2237892 was associated with increased gestational weight in American women (32). A meta-analysis of four rs2237892 studies found a positive association between the C allele and an increased risk of GDM (33). In contrast, rs2237892 was unrelated to GDM risk in Chinese, Saudi, and Caucasian populations (17, 20, 21, 34). In our study, rs2237892 did not find any association with GDM. Given the controversial outcomings of research associated with rs2237892 and GDM, a comprehensive meta-analysis is essential to investigate the impact of *KCNQ1* on GDM risk. We conducted a meta-analysis and found that rs2237892 decreased GDM risk and that the T allele was a protective gene for GDM risk. In addition, studies of the association between *KCNQ1* rs2237892 and GDM susceptibility were mainly in Asian populations and less in European populations, which may be related to the minor allele frequency of this SNP in different races. According to the NCBI SNP database search, the frequency of the minor allele is 34.5% in Asian populations and 6% in European populations.

However, our study did not find an association between rs163184 and GDM genetic susceptibility has been found in our study. The findings were also consistent with studies in Korean, Japanese, and Mexican populations. None of them found an association between rs163184 and GDM (19, 35–37). These findings suggest that rs163184 may not play a significant role in the genetic susceptibility to GDM, at least in the populations examined.

The potassium channel encoded by *KCNQ1* plays a crucial role in insulin secretion. Overexpression of the *KCNQ1* gene results in reduced glucose-induced insulin secretion (6, 7). The *KCNQ1* protein is expressed in insulin-secreting INS-1 cells and the *KCNQ1* inhibitor chromanol 293B can stimulate insulin secretion in the presence of tolbutamide (38). Thus, the *KCNQ1* regulates the membrane potential of potassium channels in pancreatic beta cells, thereby influencing insulin secretion and pancreatic beta cell function.

Additionally, all the SNPs examined in our study are located in intronic regions, which, despite being non-coding, can impact gene expression. Introns can regulate protein expression through mechanisms such as alternative splicing, positive regulation of gene expression, nonsense-mediated decay (NMD), mRNA transport, and chromatin assembly (39). The impact of SNPs on protein expression and function can affect potassium channel activity and pancreatic islet beta cell secretory function, potentially through mechanisms such as stimulation or inhibition of gene expression, post-translational modifications, or splicing in the coding region.

Although the OGTT is the primary diagnostic method for GDM, some researchers have proposed alternative diagnostic approaches, including the use of biomarkers and genetic tests, to identify GDM risk. SNPs, common in biological genomes and occurring at single nucleotide base pairs in the human DNA sequence, are novel molecular markers. With the completion of the Human Genome Project, the development of high-throughput genotyping technology, and advances in functional genomics research, an increasing number of disease-related susceptibility genes have been discovered. The study of gene polymorphisms and GDM susceptibility can provide clues for diagnosing GDM. By testing the genotypes of susceptibility genes in pregnant women, we can determine the risk of GDM and select the susceptible group for early intervention. These findings will be crucial for advancing early diagnosis and prevention strategies for GDM, in line with the United Nations Sustainable Development Goal 3 (Good Health and Well-Being), which includes maternal health and well-being.

Our study has several limitations. First, our data and analyses are incomplete. We did not analyze serum triglycerides, low-density lipoprotein (LDL), and high-density lipoprotein (HDL) to evaluate lipid metabolism in pregnant women, nor did we have data on pancreatic islets to investigate insulin resistance and pancreatic beta cell function. We lacked data on weight changes during pregnancy, preventing us from analyzing these changes. More information on lifestyle and environmental factors, such as diet and exercise, is needed. We could not analyze the combined effects of interactions between SNPs, GDM, and other factors. Second, this study is a single-center study. Our subjects were concentrated in the Shunde Women and Children’s Hospital of Guangdong Medical University, which may not represent the entire Chinese population. The conclusions of our study need to be further proven by a multicenter study across the country. Third, GDM candidate genes were selected based on T2DM risk genes. Although GDM and T2DM share similar genetic backgrounds and pathophysiological mechanisms, they are essentially two different types of diabetes. T2DM is a chronic disease, whereas GDM develops during pregnancy and resolves after birth. Our analyses lacked data and subgroups for T2DM, preventing us from comparing differences in risk effects between GDM and T2DM. Finally, we studied single genes and did not consider the combined effects of multiple genes or the interactions between genes and proteins.

In the future, we aim to conduct a multicenter collaboration, collect individuals from different regions, and analyze the combined effects of genes, lifestyle, and environment. With the aid of molecular biology experiments and biochemical analyses, we will investigate the effects of genes on protein expression and function. Through cell biology experiments and animal studies, we will further elucidate the mechanism of the KCNQ1 risk gene and diabetes susceptibility.

## 5 Conclusion

The case-control study and meta-analysis revealed that the *KCNQ1* gene is associated with GDM susceptibility, which may provide clues for predicting GDM susceptibility in Chinese populations. In particular, rs2237897 showed protection against GDM susceptibility in pregnant women aged < 30 years. The meta-analysis revealed significant associations between rs2237892 and GDM across diverse populations. The findings still need to be further confirmed, and the mechanism of its influence on GDM susceptibility needs to be clarified by further functional cell biology experiments and animal experiments.

## Data Availability

All the original data relevant to the study are included in the article and supplemental material, further inquiries can be directed to the corresponding authors.
