# Supplementary material for: Association between KCNQ1 gene polymorphisms and gestational diabetes mellitus susceptibility in a Chinese population
Source: Front Endocrinol (Lausanne). 2025 Jul 2;16:1451942. doi: 10.3389/fendo.2025.1451942 (PMC12263388; doi:10.3389/fendo.2025.1451942)
Supplement: Supplementary file 1 [file DataSheet1.docx]

**Supplementary table 1** Basic and stratified characteristics of participants with KCNQ1 rs2237897.

| Variables | NGT (n = 500) | GDM (n = 500) | t / χ^2^ | *P* |
| --- | --- | --- | --- | --- |
| Age (year) | 29 ± 4 | 31 ± 4 | -8.538 | **< 0.001** |
| Pre-BMI (kg/m^2^) | 20.53 ± 2.59 | 21.51 ± 3.10 | -5.411 | **< 0.001** |
| SBP (mmHg) | 114 ± 10 | 117 ± 11 | -3.505 | **< 0.001** |
| DBP (mmHg) | 68 ± 7 | 70 ± 8 | -3.22 | **0.001** |
| FBG (mmol/L) | 4.50 ± 0.31 | 4.82 ± 0.64 | -9.753 | **< 0.001** |
| 1h-PG (mmol/L) | 7.66 ± 1.27 | 10.17 ± 1.60 | -26.187 | **< 0.001** |
| 2h-PG (mmol/L) | 6.69 ± 0.99 | 8.91 ± 1.59 | -25.847 | **< 0.001** |
| Age (year) |  |  | 49.289 | **< 0.001** |
| < 30 | 303 (0.606) | 192 (0.384) |  |  |
| ≥ 30 | 197 (0.394) | 308 (0.616) |  |  |
| Pre-BMI (kg/m^2^) |  |  | 27.411 | **< 0.001** |
| < 18.5 | 94 (0.188) | 67 (0.134) |  |  |
| 18.5 ≤ pre-BMI < 24 | 364 (0.728) | 336 (0.672) |  |  |
| ≥24 | 42 (0.084) | 97 (0.194) |  |  |
| Parity (n) |  |  | 8.875 | **0.003** |
| 0 | 257 (0.514) | 210 (0.42) |  |  |
| ≥ 1 | 243 (0.486) | 290 (0.58) |  |  |

NGT, normal glucose tolerance; GDM, Gestational diabetes mellitus; Pre-BMI, pre-gestational body mass index; SBP, systolic blood pressure; DBP, diastolic blood pressure; FBG, fasting blood glucose level; 1h-PG, 1 hour blood glucose level; 2h-PG, 2 hour blood glucose level; bold values indicate the *P* < 0.05.

**Supplementary table 2** SNPs MAF and HWE test in the controls.

| SNPs | Min / Maj | Chr. position | Region | MAF | HWE(control) |
| --- | --- | --- | --- | --- | --- |
| rs2237897 | T / C | chr11: 2837316 | intron | 0.287 | 0.999 |
| rs163184 | G / T | chr11: 2825839 | intron | 0.434 | 0.13 |
| rs151290 | A / C | chr11: 2800385 | intron | 0.375 | 0.988 |
| rs2237892 | T / C | chr11: 2818521 | intron | 0.295 | 0.993 |

SNPs, single nucleotide polymorphisms; Min, minor allele; Maj, major allele; MAF, frequency of minor allele; HWE, Hardy–Weinberg equilibrium.

**Supplementary table 3** The associations between KCNQ1 gene and GDM risk in age ≥ 30 years subjects.

| Model | Controls (%) | Cases (%) | Crude OR (95 % CI) | Crude *P* | Adjusted OR (95 % CI) | Adjusted *P* |
| --- | --- | --- | --- | --- | --- | --- |
| rs2237897 | | | | | | |
| Codominant model | | | | | | |
| CC | 90 (0.457) | 151 (0.490) | 1 (Reference) |  | 1 (Reference) |  |
| CT | 90 (0.457) | 142 (0.461) | 0.940 (0.649-1.363) | 0.746 | 0.901 (0.615-1.320) | 0.593 |
| TT | 17 (0.086) | 15 (0.049) | 0.526 (0.250-1.104) | 0.089 | 0.541 (0.251-1.163) | 0.116 |
| Dominant Model | | | | | | |
| CC | 90 (0.457) | 151 (0.490) | 1 (Reference) |  | 1 (Reference) |  |
| CT+TT | 107 (0.543) | 157 (0.510) | 0.875 (0.611-1.252) | 0.464 | 0.845 (0.584-1.221) | 0.370 |
| Recessive Model | | | | | | |
| CT+CC | 180 (0.914) | 293 (0.951) | 1 (Reference) |  | 1 (Reference) |  |
| TT | 17 (0.086) | 15 (0.049) | 0.542 (0.264-1.112) | 0.095 | 0.577 (0.278-1.199) | 0.141 |
| Overdominant model | | | | | | |
| TT+CC | 107 (0.543) | 166 (0.539) | 1 (Reference) |  | 1 (Reference) |  |
| CT | 90 (0.457) | 142 (0.461) | 1.017 (0.710-1.456) | 0.927 | 0.970 (0.671-1.403) | 0.872 |
| Aelle model | | | | | | |
| C | 270 (0.685) | 444 (0.721) | 1 (Reference) |  | 1 (Reference) |  |
| T | 124 (0.315) | 172 (0.279) | 0.844 (0.640-1.112) | 0.227 | 0.834 (0.628-1.106) | 0.207 |
| rs163184 | | | | | | |
| Codominant model | | | | | | |
| TT | 59 (0.298) | 97 (0.315) | 1 (Reference) |  | 1 (Reference) |  |
| GT | 107 (0.540) | 157 (0.510) | 0.892 (0.594-1.340) | 0.583 | 0.892 (0.586-1.359) | 0.595 |
| GG | 32 (0.162) | 54 (0.175) | 1.026 (0.596-1.768) | 0.925 | 1.054 (0.605-1.835) | 0.853 |
| Dominant Model | | | | | | |
| TT | 59 (0.298) | 97 (0.315) | 1 (Reference) |  | 1 (Reference) |  |
| GT+GG | 139 (0.702) | 211 (0.685) | 0.923 (0.626-1.361) | 0.687 | 0.928 (0.623-1.383) | 0.714 |
| Recessive Model | | | | | | |
| GT+TT | 166 (0.838) | 254 (0.825) | 1 (Reference) |  | 1 (Reference) |  |
| GG | 32 (0.162) | 54 (0.175) | 1.103 (0.683-1.781) | 0.689 | 1.122 (0.685-1.839) | 0.647 |
| Overdominant model | | | | | | |
| GG+TT | 91 (0.460) | 151 (0.490) | 1 (Reference) |  | 1 (Reference) |  |
| GT | 107 (0.540) | 157 (0.510) | 0.884 (0.618-1.265) | 0.500 | 0.880 (0.609-1.271) | 0.495 |
| Aelle model | | | | | | |
| T | 225 (0.568) | 351 (0.570) | 1 (Reference) |  | 1 (Reference) |  |
| G | 171 (0.432) | 265 (0.430) | 0.993 (0.770-1.282) | 0.959 | 1.000 (0.770-1.299) | 0.997 |
| rs151290 | | | | | | |
| Codominant model | | | | | | |
| CC | 70 (0.354) | 129 (0.419) | 1 (Reference) |  | 1 (Reference) |  |
| CA | 98 (0.495) | 130 (0.422) | 0.720 (0.487-1.065) | 0.100 | 0.714 (0.478-1.065) | 0.099 |
| AA | 30 (0.151) | 49 (0.159) | 0.886 (0.517-1.520) | 0.661 | 0.918 (0.523-1.613) | 0.767 |
| Dominant Model | | | | | | |
| CC | 70 (0.354) | 129 (0.419) | 1 (Reference) |  | 1 (Reference) |  |
| CA+AA | 128 (0.646) | 179 (0.581) | 0.759 (0.525-1.097) | 0.143 | 0.756 (0.518-1.103) | 0.147 |
| Recessive Model | | | | | | |
| CA+CC | 168 (0.849) | 259 (0.841) | 1 (Reference) |  | 1 (Reference) |  |
| AA | 30 (0.151) | 49 (0.159) | 1.059 (0.646-1.736) | 0.819 | 1.085 (0.654-1.800) | 0.752 |
| Overdominant model | | | | | | |
| AA+CC | 100 (0.505) | 178 (0.578) | 1 (Reference) |  | 1 (Reference) |  |
| CA | 98 (0.495) | 130 (0.422) | 0.745 (0.521-1.067) | 0.108 | 0.800 (0.547-1.169) | 0.248 |
| Aelle model | | | | | | |
| C | 238 (0.601) | 388 (0.630) | 1 (Reference) |  | 1 (Reference) |  |
| A | 158 (0.399) | 228 (0.370) | 0.885 (0.683-1.147) | 0.356 | 0.890 (0.682-1.160) | 0.387 |
| rs2237892 | | | | | | |
| Codominant model | | | | | | |
| CC | 92 (0.465) | 151 (0.490) | 1 (Reference) |  | 1 (Reference) |  |
| TC | 90 (0.454) | 133 (0.432) | 0.900 (0.620-1.307) | 0.581 | 0.889 (0.607-1.303) | 0.546 |
| TT | 16 (0.081) | 24 (0.078) | 0.914 (0.461-1.810) | 0.796 | 0.921 (0.455-1.864) | 0.819 |
| Dominant Model | | | | | | |
| CC | 92 (0.465) | 151 (0.490) | 1 (Reference) |  | 1 (Reference) |  |
| CT+TT | 106 (0.535) | 157 (0.510) | 0.902 (0.631-1.290) | 0.574 | 0.894 (0.620-1.290) | 0.549 |
| Recessive Model | | | | | | |
| CT+CC | 182 (0.919) | 284 (0.922) | 1 (Reference) |  | 1 (Reference) |  |
| TT | 16 (0.081) | 24 (0.078) | 0.961 (0.497-1.859) | 0.907 | 1.001 (0.510-1.965) | 0.998 |
| Overdominant model | | | | | | |
| TT+CC | 108 (0.546) | 175 (0.568) | 1 (Reference) |  | 1 (Reference) |  |
| CT | 90 (0.454) | 133 (0.432) | 0.912 (0.637-1.306) | 0.615 | 0.893 (0.618-1.290) | 0.546 |
| Aelle model | | | | | | |
| C | 274 (0.692) | 435 (0.706) | 1 (Reference) |  | 1 (Reference) |  |
| T | 122 (0.308) | 181 (0.294) | 0.935 (0.710-1.230) | 0.629 | 0.936 (0.707-1.240) | 0.646 |

Adjusted *P* value calculated by logistic regression with adjustment for age, pre-BMI, SBP, DBP and parity. The control group for SNP rs2237897 had 197 participants in the age ≥ 30 years subgroup, and the control group for SNP rs163184, rs151290 and rs2237892 had 198 participants in the age ≥ 30 years subgroup. The case groups for SNP rs2237897, rs163184, rs151290 and rs2237892 all had 308 participants in the age ≥ 30 years subgroup.

**Supplementary table 4** The associations between KCNQ1 gene and GDM risk in pre-BMI < 18.5 kg/m^2^ subjects.

| Model | Controls (%) | Cases (%) | Crude OR (95 % CI) | Crude *P* | Adjusted OR (95 % CI) | Adjusted *P* |
| --- | --- | --- | --- | --- | --- | --- |
| rs2237897 | | | | | | |
| Codominant model | | | | | | |
| CC | 49 (0.521) | 39 (0.582) | 1 (Reference) |  | 1 (Reference) |  |
| CT | 35 (0.372) | 23 (0.343) | 0.826 (0.421-1.619) | 0.577 | 0.763 (0.374-1.559) | 0.459 |
| TT | 10 (0.107) | 5 (0.075) | 0.628 (0.198-1.990) | 0.429 | 0.600 (0.169-2.126) | 0.429 |
| Dominant Model | | | | | | |
| CC | 49 (0.521) | 39 (0.582) | 1 (Reference) |  | 1 (Reference) |  |
| CT+TT | 45 (0.479) | 28 (0.418) | 0.782 (0.416-1.471) | 0.445 | 0.776 (0.400-1.508) | 0.455 |
| Recessive Model | | | | | | |
| CT+CC | 84 (0.893) | 62 (0.925) | 1 (Reference) |  | 1 (Reference) |  |
| TT | 10 (0.107) | 5 (0.075) | 0.677 (0.220-2.082) | 0.497 | 0.777 (0.233-2.590) | 0.682 |
| Overdominant model | | | | | | |
| TT+CC | 59 (0.628) | 44 (0.657) | 1 (Reference) |  | 1 (Reference) |  |
| CT | 35 (0.372) | 23 (0.343) | 0.881 (0.458-1.697) | 0.705 | 0.829 (0.416-1.651) | 0.593 |
| Aelle model | | | | | | |
| C | 133 (0.707) | 101 (0.754) | 1 (Reference) |  | 1 (Reference) |  |
| T | 55 (0.293) | 33 (0.246) | 0.790 (0.478-1.307) | 0.359 | 0.813 (0.480-1.376) | 0.440 |
| rs163184 | | | | | | |
| Codominant model | | | | | | |
| TT | 28 (0.295) | 17 (0.254) | 1 (Reference) |  | 1 (Reference) |  |
| GT | 53 (0.558) | 36 (0.537) | 1.119 (0.536-2.337) | 0.765 | 1.134 (0.496-2.590) | 0.766 |
| GG | 14 (0.147) | 14 (0.209) | 1.647 (0.634-4.280) | 0.306 | 1.046 (0.348-3.140) | 0.936 |
| Dominant Model | | | | | | |
| TT | 28 (0.295) | 17 (0.254) | 1 (Reference) |  | 1 (Reference) |  |
| GT+GG | 67 (0.705) | 50 (0.746) | 1.229 (0.607-2.488) | 0.566 | 1.012 (0.476-2.152) | 0.975 |
| Recessive Model | | | | | | |
| GT+TT | 81 (0.853) | 53 (0.791) | 1 (Reference) |  | 1 (Reference) |  |
| GG | 14 (0.147) | 14 (0.209) | 1.528 (0.675-3.462) | 0.309 | 1.471 (0.620-3.491) | 0.381 |
| Overdominant model | | | | | | |
| GG+TT | 42 (0.442) | 31 (0.463) | 1 (Reference) |  | 1 (Reference) |  |
| GT | 53 (0.558) | 36 (0.537) | 0.920 (0.491-1.725) | 0.795 | 0.798 (0.405-1.572) | 0.514 |
| Aelle model | | | | | | |
| T | 109 (0.574) | 70 (0.522) | 1 (Reference) |  | 1 (Reference) |  |
| G | 81 (0.426) | 64 (0.478) | 1.230 (0.789-1.919) | 0.361 | 1.123 (0.705-1.790) | 0.625 |
| rs151290 | | | | | | |
| Codominant model | | | | | | |
| CC | 38 (0.4) | 30 (0.448) | 1 (Reference) |  | 1 (Reference) |  |
| CA | 43 (0.453) | 29 (0.433) | 0.854 (0.436-1.672) | 0.646 | 0.842 (0.410-1.728) | 0.639 |
| AA | 14 (0.147) | 8 (0.119) | 0.724 (0.268-1.951) | 0.523 | 0.712 (0.260-1.954) | 0.510 |
| Dominant Model | | | | | | |
| CC | 38 (0.4) | 30 (0.448) | 1 (Reference) |  | 1 (Reference) |  |
| CA+AA | 57 (0.6) | 37 (0.552) | 0.822 (0.437-1.548) | 0.544 | 0.842 (0.436-1.627) | 0.609 |
| Recessive Model | | | | | | |
| CA+CC | 81 (0.853) | 59 (0.881) | 1 (Reference) |  | 1 (Reference) |  |
| AA | 14 (0.147) | 8 (0.119) | 0.785 (0.309-1.991) | 0.609 | 0.813 (0.308-2.146) | 0.675 |
| Overdominant model | | | | | | |
| AA+CC | 52 (0.547) | 38 (0.567) | 1 (Reference) |  | 1 (Reference) |  |
| CA | 43 (0.453) | 29 (0.433) | 0.923 (0.492-1.733) | 0.803 | 0.929 (0.482-1.791) | 0.825 |
| Aelle model | | | | | | |
| C | 119 (0.626) | 89 (0.664) | 1 (Reference) |  | 1 (Reference) |  |
| A | 71 (0.374) | 45 (0.336) | 0.847 (0.533-1.347) | 0.484 | 0.865 (0.533-1.404) | 0.558 |
| rs2237892 | | | | | | |
| Codominant model | | | | | | |
| CC | 44 (0.463) | 36 (0.537) | 1 (Reference) |  | 1 (Reference) |  |
| TC | 40 (0.421) | 25 (0.373) | 0.764 (0.392-1.487) | 0.428 | 0.711 (0.350-1.445) | 0.346 |
| TT | 11 (0.116) | 6 (0.09) | 0.667 (0.225-1.979) | 0.465 | 0.808 (0.249-2.618) | 0.722 |
| Dominant Model | | | | | | |
| CC | 44 (0.463) | 36 (0.537) | 1 (Reference) |  | 1 (Reference) |  |
| CT+TT | 51 (0.537) | 31 (0.463) | 0.743 (0.397-1.391) | 0.353 | 0.775 (0.402-1.495) | 0.447 |
| Recessive Model | | | | | | |
| CT+CC | 84 (0.884) | 61 (0.91) | 1 (Reference) |  | 1 (Reference) |  |
| TT | 11 (0.116) | 6 (0.09) | 0.751 (0.263-2.142) | 0.592 | 1.017 (0.329-3.144) | 0.976 |
| Overdominant model | | | | | | |
| TT+CC | 55 (0.579) | 42 (0.627) | 1 (Reference) |  | 1 (Reference) |  |
| CT | 40 (0.421) | 25 (0.373) | 0.818 (0.431-1.554) | 0.540 | 0.760 (0.387-1.495) | 0.427 |
| Aelle model | | | | | | |
| C | 128 (0.674) | 97 (0.724) | 1 (Reference) |  | 1 (Reference) |  |
| T | 62 (0.326) | 37 (0.276) | 0.787 (0.485-1.279) | 0.335 | 0.862 (0.518-1.433) | 0.566 |

Pre-BMI, pre-gestational body mass index; adjusted *P* value calculated by logistic regression with adjustment for age, pre-BMI, SBP, DBP and parity. The control group for SNP rs2237897 had 94 participants in the pre-BMI < 18.5 kg/m^2^ subgroup, and the control group for SNP rs163184, rs151290 and rs2237892 had 95 participants in the pre-BMI < 18.5 kg/m^2^ subgroup. The case groups for SNP rs2237897, rs163184, rs151290 and rs2237892 all had 67 participants in the pre-BMI > 18.5 kg/m^2^ subgroup.

**Supplementary table 5** The associations between KCNQ1 gene and GDM risk in 18.5kg/m^2^ ≤ pre-BMI < 24 kg/m^2^ subjects.

| Model | Controls (%) | Cases (%) | Crude OR (95 % CI) | Crude *P* | Adjusted OR (95 % CI) | Adjusted *P* |
| --- | --- | --- | --- | --- | --- | --- |
| rs2237897 | | | | | | |
| Codominant model | | | | | | |
| CC | 179 (0.492) | 175 (0.521) | 1 (Reference) |  | 1 (Reference) |  |
| CT | 153 (0.420) | 134 (0.399) | 0.896 (0.656-1.224) | 0.489 | 0.780 (0.561-1.084) | 0.138 |
| TT | 32 (0.088) | 27 (0.080) | 0.863 (0.496-1.500) | 0.602 | 0.834 (0.471-1.475) | 0.532 |
| Dominant Model | | | | | | |
| CC | 179 (0.492) | 175 (0.521) | 1 (Reference) |  | 1 (Reference) |  |
| CT+TT | 185 (0.508) | 161 (0.479) | 0.890 (0.662-1.198) | 0.442 | 0.792 (0.580-1.081) | 0.142 |
| Recessive Model | | | | | | |
| CT+CC | 332 (0.912) | 309 (0.920) | 1 (Reference) |  | 1 (Reference) |  |
| TT | 32 (0.088) | 27 (0.080) | 0.907 (0.531-1.548) | 0.719 | 0.929 (0.534-1.616) | 0.794 |
| Overdominant model | | | | | | |
| TT+CC | 211 (0.580) | 202 (0.601) | 1 (Reference) |  | 1 (Reference) |  |
| CT | 153 (0.420) | 134 (0.399) | 0.915 (0.677-1.237) | 0.563 | 0.804 (0.586-1.105) | 0.178 |
| Aelle model | | | | | | |
| C | 511 (0.702) | 484 (0.720) | 1 (Reference) |  | 1 (Reference) |  |
| T | 217 (0.298) | 188 (0.280) | 0.915 (0.726-1.153) | 0.450 | 0.857 (0.673-1.091) | 0.210 |
| rs163184 | | | | | | |
| Codominant model | | | | | | |
| TT | 111 (0.304) | 103 (0.307) | 1 (Reference) |  | 1 (Reference) |  |
| GT | 194 (0.532) | 170 (0.506) | 0.944 (0.673-1.324) | 0.740 | 0.950 (0.667-1.353) | 0.776 |
| GG | 60 (0.164) | 63 (0.187) | 1.132 (0.726-1.764) | 0.585 | 1.227 (0.768-1.961) | 0.392 |
| Dominant Model | | | | | | |
| TT | 111 (0.304) | 103 (0.307) | 1 (Reference) |  | 1 (Reference) |  |
| GT+GG | 254 (0.696) | 233 (0.693) | 0.989 (0.717-1.364) | 0.944 | 1.012 (0.723-1.416) | 0.946 |
| Recessive Model | | | | | | |
| GT+TT | 305 (0.836) | 273 (0.813) | 1 (Reference) |  | 1 (Reference) |  |
| GG | 60 (0.164) | 63 (0.187) | 1.173 (0.795-1.732) | 0.422 | 1.271 (0.843-1.916) | 0.252 |
| Overdominant model | | | | | | |
| GG+TT | 171 (0.468) | 166 (0.494) | 1 (Reference) |  | 1 (Reference) |  |
| GT | 194 (0.532) | 170 (0.506) | 0.903 (0.671-1.214) | 0.499 | 0.881 (0.646-1.201) | 0.422 |
| Aelle model | | | | | | |
| T | 416 (0.570) | 376 (0.559) | 1 (Reference) |  | 1 (Reference) |  |
| G | 314 (0.430) | 296 (0.441) | 1.043 (0.844-1.288) | 0.696 | 1.077 (0.864-1.343) | 0.508 |
| rs151290 | | | | | | |
| Codominant model | | | | | | |
| CC | 139 (0.381) | 139 (0.414) | 1 (Reference) |  | 1 (Reference) |  |
| CA | 172 (0.471) | 144 (0.428) | 0.837 (0.606-1.156) | 0.281 | 0.806 (0.575-1.129) | 0.210 |
| AA | 54 (0.148) | 53 (0.158) | 0.981 (0.628-1.533) | 0.935 | 0.955 (0.592-1.539) | 0.849 |
| Dominant Model | | | | | | |
| CC | 139 (0.381) | 139 (0.414) | 1 (Reference) |  | 1 (Reference) |  |
| CA+AA | 226 (0.619) | 197 (0.586) | 0.872 (0.644-1.180) | 0.374 | 0.838 (0.610-1.149) | 0.273 |
| Recessive Model | | | | | | |
| CA+CC | 311 (0.852) | 283 (0.842) | 1 (Reference) |  | 1 (Reference) |  |
| AA | 54 (0.148) | 53 (0.158) | 1.079 (0.715-1.628) | 0.719 | 1.054 (0.686-1.620) | 0.809 |
| Overdominant model | | | | | | |
| AA+CC | 193 (0.529) | 192 (0.572) | 1 (Reference) |  | 1 (Reference) |  |
| CA | 172 (0.471) | 144 (0.428) | 0.842 (0.625-1.134) | 0.257 | 0.819 (0.600-1.119) | 0.210 |
| Aelle model | | | | | | |
| C | 450 (0.616) | 422 (0.628) | 1 (Reference) |  | 1 (Reference) |  |
| A | 280 (0.384) | 250 (0.372) | 0.952 (0.767-1.182) | 0.656 | 0.928 (0.740-1.162) | 0.513 |
| rs2237892 | | | | | | |
| Codominant model | | | | | | |
| CC | 181 (0.496) | 176 (0.524) | 1 (Reference) |  | 1 (Reference) |  |
| TC | 152 (0.416) | 129 (0.384) | 0.873 (0.638-1.194) | 0.395 | 0.782 (0.563-1.087) | 0.144 |
| TT | 32 (0.088) | 31 (0.092) | 0.996 (0.583-1.702) | 0.989 | 0.931 (0.533-1.626) | 0.802 |
| Dominant Model | | | | | | |
| CC | 181 (0.496) | 176 (0.524) | 1 (Reference) |  | 1 (Reference) |  |
| CT+TT | 184 (0.504) | 160 (0.476) | 0.894 (0.665-1.203) | 0.460 | 0.810 (0.594-1.106) | 0.185 |
| Recessive Model | | | | | | |
| CT+CC | 333 (0.912) | 305 (0.908) | 1 (Reference) |  | 1 (Reference) |  |
| TT | 32 (0.088) | 31 (0.092) | 1.058 (0.630-1.775) | 0.832 | 1.054 (0.616-1.802) | 0.848 |
| Overdominant model | | | | | | |
| TT+CC | 213 (0.584) | 207 (0.616) | 1 (Reference) |  | 1 (Reference) |  |
| CT | 152 (0.416) | 129 (0.384) | 0.873 (0.645-1.182) | 0.380 | 0.789 (0.574-1.083) | 0.143 |
| Aelle model | | | | | | |
| C | 514 (0.704) | 481 (0.716) | 1 (Reference) |  | 1 (Reference) |  |
| T | 216 (0.296) | 191 (0.284) | 0.945 (0.750-1.190) | 0.631 | 0.890 (0.700-1.133) | 0.345 |

Pre-BMI, pre-gestational body mass index; adjusted *P* value calculated by logistic regression with adjustment for age, pre-BMI, SBP, DBP and parity. The control group for SNP rs2237897 had 364 participants in the 18.5kg/m^2^ ≤ pre-BMI < 24 kg/m^2^ subgroup, and the control group for SNP rs163184, rs151290 and rs2237892 had 365 participants in the 18.5kg/m^2^ ≤ pre-BMI < 24 kg/m^2^ subgroup. The case groups for SNP rs2237897, rs163184, rs151290 and rs2237892 all had 336 participants in the 18.5kg/m^2^ ≤ pre-BMI < 24 kg/m^2^ subgroup.

**Supplementary table 6** Haplotype analysis of the KCNQ1 rs163184, rs151290 and rs2237892 and GDM risk.

| Haplotype | Case | Control | X^2^ | *P* | OR (95 % CI) |
| --- | --- | --- | --- | --- | --- |
| TAT | 262 | 292 | 2.082 | 0.148 | 0.865 (0.711-1.053) |
| GCC | 393 | 390 | 0.043 | 0.834 | 1.019 (0.851-1.219) |
| TCC | 222 | 212 | 0.347 | 0.555 | 1.066 (0.861-1.318) |
| GAC | 46 | 40 | 0.462 | 0.496 | 1.162 (0.753-1.791) |
| TAC | 57 | 53 | 0.171 | 0.678 | 1.084 (0.738-1.593) |

Haplotype with frequency ＜0.03 are ignored.

**Supplementary table 7** Association between polymorphic genotypes of SNPs and blood glucose levels, neonatal weight, and Gestation age.

| genotype | FBG (mmol/L) | 1h-PG (mmol/L) | 2h-PG (mmol/L) | Neonatal weight (g) | Gestation age (week) |
| --- | --- | --- | --- | --- | --- |
| rs2237897 |  |  |  |  |  |
| CC | 4.70 ± 0.63 | 9.03 ± 1.95 | 7.91 ± 1.75 | 3183.33 ± 383.96 | 39.02 ± 1.29 |
| CT | 4.64 ± 0.42 | 9.93 ± 1.92 | 7.89 ± 1.76 | 3205.54 ± 376.13 | 38.91 ± 2.47 |
| TT | 4.62 ± 0.44 | 8.72 ± 1.72 | 7.66 ± 1.59 | 3191.90 ± 359.03 | 39.17 ± 1.40 |
| F | 1.312 | 0.969 | 0.691 | 0.389 | 0.799 |
| *P* | 0.270 | 0.380 | 0.501 | 0.678 | 0.45 |
| rs163184 |  |  |  |  |  |
| TT | 4.65 ± 0.45 | 8.96 ± 1.90 | 7.91 ± 1.78 | 3218.22 ± 388.34 | 38.99 ± 2.22 |
| TG | 4.68 ± 0.62 | 9.02 ± 1.97 | 7.90 ± 1.78 | 3175.53 ± 372.33 | 39.04 ± 1.43 |
| GG | 4.67 ± 0.41 | 9.03 ± 1.80 | 7.74 ± 1.56 | 3204.68 ± 382.04 | 38.82 ± 2.32 |
| F | 0.447 | 0.081 | 0.573 | 1.313 | 0.939 |
| *P* | 0.64 | 0.922 | 0.564 | 0.27 | 0.391 |
| rs151290 |  |  |  |  |  |
| CC | 4.68 ± 0.64 | 9.10 ± 1.97 | 7.91 ± 1.81 | 3180.11 ± 385.38 | 39.01 ± 1.25 |
| AC | 4.65 ± 0.45 | 8.89 ± 1.88 | 7.83 ± 1.71 | 3198.36 ± 373.74 | 39.00 ± 1.96 |
| AA | 4.68 ± 0.45 | 9.06 ± 1.87 | 7.92 ± 1.64 | 3214.14 ± 378.56 | 38.88 ± 2.74 |
| F | 0.290 | 1.211 | 0.266 | 0.513 | 0.333 |
| *P* | 0.748 | 0.298 | 0.766 | 0.599 | 0.717 |
| rs2237892 |  |  |  |  |  |
| CC | 4.69 ± 0.61 | 9.03 ± 1.95 | 7.87 ± 1.75 | 3184.49 ± 387.16 | 38.97 ± 1.76 |
| TC | 4.65 ± 0.45 | 9.01 ± 1.93 | 7.91 ± 1.76 | 3195.91 ± 370.00 | 39.07 ± 1.50 |
| TT | 4.64 ± 0.44 | 8.79 ± 1.68 | 7.75 ± 1.62 | 3231.20 ± 374.83 | 38.74 ± 3.32 |
| F | 0.660 | 0.582 | 0.327 | 0.605 | 1.260 |
| *P* | 0.517 | 0.559 | 0.721 | 0.546 | 0.284 |

FBG, fasting blood glucose level; 1h-PG, 1 hour blood glucose level; 2h-PG, 2 hour blood glucose level.

**Supplementary table 8** Characteristics of the study included in the meta-analysis.

| SNPs | Study | Year | Ethnicity | GDM criteria | Genotype | | | | | | HWE |
| --- | --- | --- | --- | --- | --- | --- | --- | --- | --- | --- | --- |
|  |  |  |  |  | Case | | | Control | | |  |
|  |  |  |  |  | AA | Aa | aa | AA | Aa | aa |  |
| rs2237892 | Wu et al.(Our study) | 2024 | Chinese | IADPSG | 263 | 192 | 45 | 240 | 215 | 47 | > 0.05 |
|  | Alshammary et al. | 2023 | Saudi | ADA | 86 | 10 | 4 | 80 | 17 | 3 | > 0.05 |
|  | Majcher et al. | 2022 | Caucasian | IADPSG | 172 | 30 | 2 | 181 | 24 | 2 | > 0.05 |
|  | Kasuga et al. | 2017 | Japanese | IADPSG | 68 | 80 | 23 | 57 | 57 | 14 | > 0.05 |
|  | Ao et al. | 2015 | Chinese | IADPSG | 323 | 206 | 33 | 202 | 201 | 50 | > 0.05 |
|  | Kwak et al. | 2010 | Korean | GCT, OGTT | 367 | 390 | 96 | 235 | 289 | 100 | > 0.05 |
|  | Zhou et al. | 2009 | Chinese | GCT, OGTT | 253 | 227 | 40 | 418 | 404 | 94 | > 0.05 |
| rs151290 | Wu et al.(Our study) | 2024 | Chinese | IADPSG | 213 | 208 | 79 | 190 | 239 | 73 | > 0.05 |
|  | Majcher et al. | 2022 | Caucasian | IADPSG | 113 | 79 | 12 | 117 | 76 | 14 | > 0.05 |
|  | Chon et al. | 2012 | Korean | GCT, OGTT | 30 | 46 | 16 | 17 | 18 | 6 | > 0.05 |

IADPSG, Implementation of the International Association of Diabetes and Pregnancy Study Groups; ADA, American Diabetes Association; OGTT, oral glucose tolerance test; GCT, glucose challenge test; AA, homozygous wild type; Aa, heterozygous genotype; aa, homozygous variant; HWE, Hardy–Weinberg equilibrium; SNPs, single nucleotide polymorphisms.


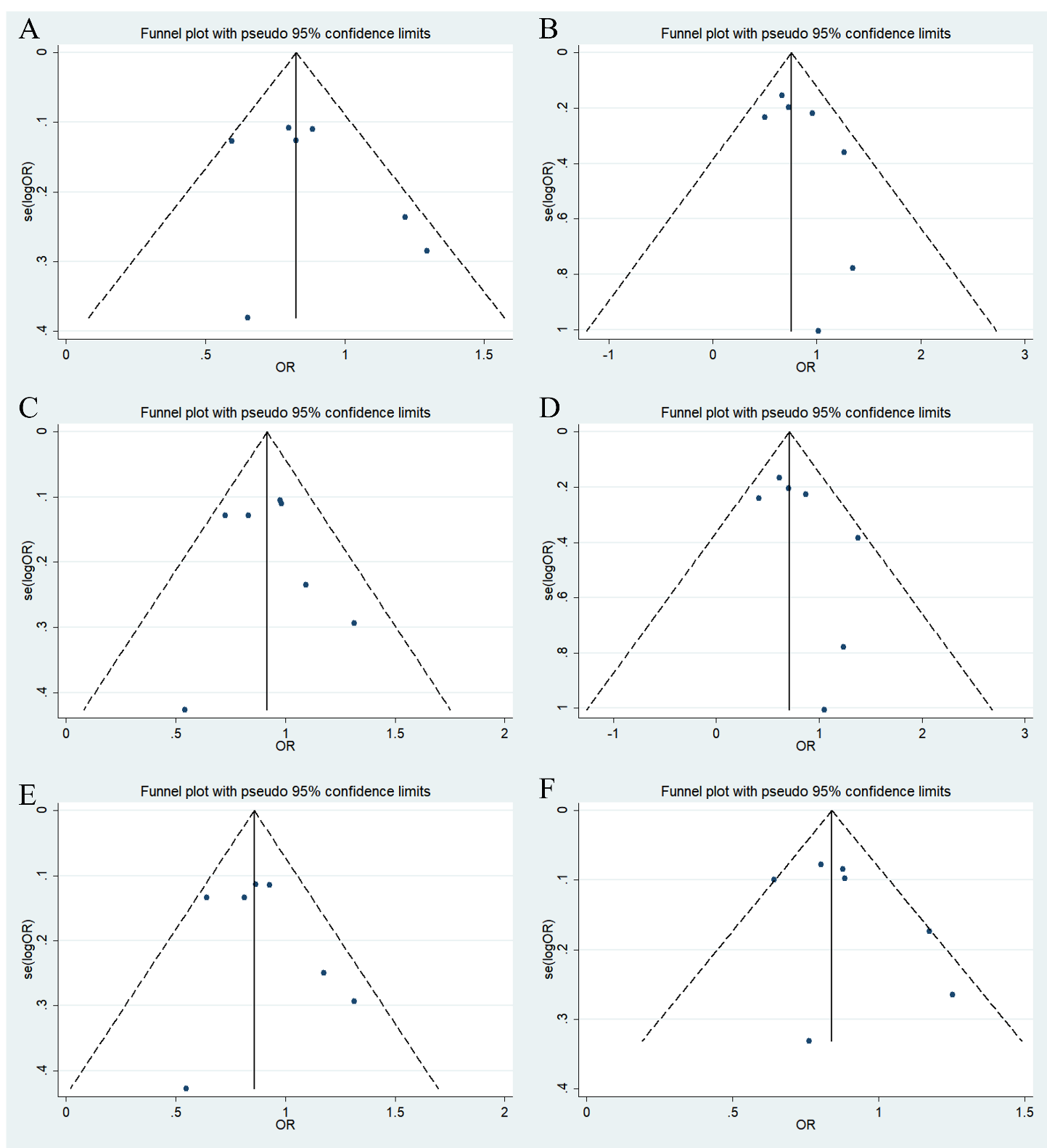


**Supplementary figure 1** Funnel plots for publication biases of different genetic models in rs2237892. (A) dominant model, TT+CT vs. CC; (B) recessive model, TT vs. CT+CC; (C) overdominant model, CT vs. TT+CC; (D) codominant homozygous model, TT vs. CC; (E) codominant heterozygous model, CT vs. CC; (F) allele model, T vs. C. Indicating publication bias based on symmetry.


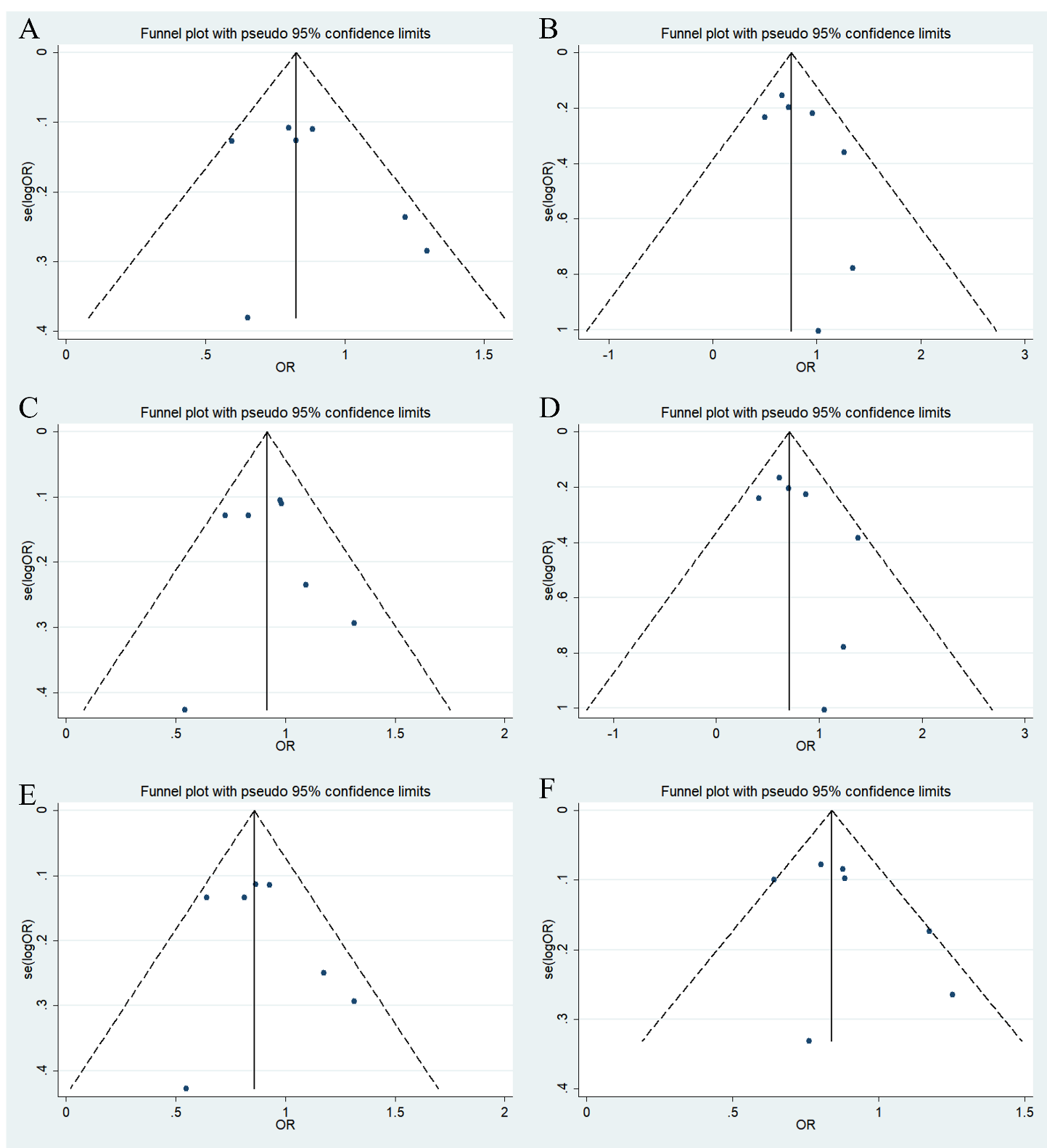


**Supplementary figure 2** Funnel plots for publication biases of different genetic models in rs151290. (A) dominant model, AA+CA vs. CC; (B) recessive model, AA vs. CA+CC; (C) overdominant model, CA vs. AA +CC; (D) codominant homozygous model, AA vs. CC; (E) codominant heterozygous model, CA vs. CC; (F) allele model, A vs. C. Indicating publication bias based on symmetry.
